# Influence laws of air gap structure manipulation of covalent organic frameworks on dielectric properties and exciton effects for photopolymerization†

**DOI:** 10.1039/d3sc01719b

**Published:** 2023-07-05

**Authors:** Hongjie Yang, Zhen Lu, Xiangyu Yin, Shengjin Wu, Linxi Hou

**Affiliations:** a Department of Materials-Oriented Chemical Engineering, School of Chemical Engineering, Fuzhou University Fuzhou 350116 P. R. China lxhou@fzu.edu.cn; b Qingyuan Innovation Laboratory Quanzhou 362801 P. R. China; c Fujian Key Laboratory of Advanced Manufacturing Technology of Specialty Chemicals, Fuzhou University Fuzhou 350116 P. R. China

## Abstract

Boosting the dissociation of excitons is essential to enhance the photocatalytic efficiency. However, the relationship between the structure of the catalyst and the exciton effect on the photocatalytic activity is still unclear as the main problem. Here, it is proposed that as a descriptive factor, an experimentally measurable dielectric constant (*ε*_r_) is available to quantitatively describe its relationship with exciton binding energy (*E*_b_) and photocatalytic activity. With tuning the linker of covalent organic frameworks (COFs), the “air gap” structure is oriented to shrink, leading to an increased *ε*_r_ of COFs and a lower *E*_b_ to facilitate exciton dissociation. Meanwhile, taking “water-/oxygen-fueled” photo-induced electron transfer reversible addition–fragmentation chain transfer (PET-RAFT) polymerization as a demonstration platform, it can be seen that COFs with a small “air gap” structure have relatively superior photocatalytic activity. This provides important implications for the evolution of efficient photocatalysts.

## Abstract 2

The dielectric constant is acted as a descriptor to help reverse the rational design of COF-based photocatalysis with low exciton binding energy to enhance the photocatalytic activity.

## Graphical abstract

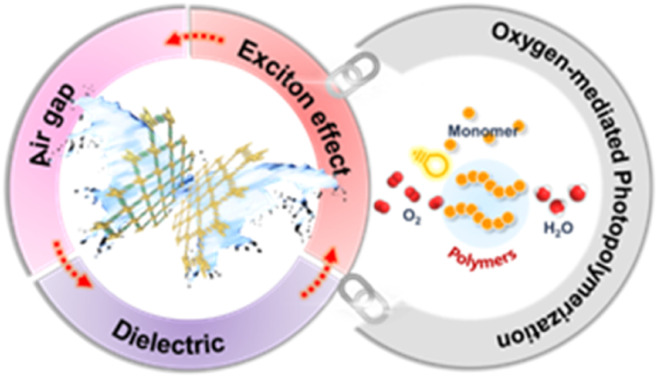

## Introduction

Photocatalysis has distinguished itself as among the most prospective orientations for sustainable solar energy conversion, with its capacity to harness light energy and to accomplish deep reactions at room temperature.^1,2^ However, exciton effect remains a major factor to restrict the photocatalytic activity.^3,4^ Photocatalysts absorb photon energy to generate “hot” electron–hole pairs, which are bound by Coulomb forces that suppress their dissociation to form free carriers, thus reducing the conversion efficiency of light energy.^5–10^ Consequently, facilitating the dissociation of excitons is critical to improving the photocatalyst activity.

Covalent organic frameworks (COFs) comprise an attractive category of crystalline organic polymer photocatalysts with pre-programmed structures capable of fulfilling functionally targeted design requirements.^11,12^ COF-based photocatalysts have made substantial progress in promoting exciton dissociation, such as donor–acceptor strategies,^13^ heterojunctions,^14^ and introduction of electron sacrificial agents.^15^ However, relatively few reports have proposed theories and methods for the inverse on-demand design of COF-based photocatalytic materials based on the correlation between the catalyst structure and photocatalytic activity. Compared with the high dielectric constant (*ε*_r_) of inorganic semiconductor photocatalysts, the *ε*_r_ of organic polymer photocatalysts is small, and their strong binding may even trigger the decay of excitons.^10^ In particular, the interlocking iso-network structure and low-polarity periodic skeleton of COFs make the dissociation of excitons difficult.

The *ε*_r_ is directly related to the polarizability of the material and depends heavily on the chemical structure of the material.^16^ According to the Wannier–Mott model:^17^*E*_b_ = *E*_H_m*/*ε*_r_^2^ (where *E*_b_ is the exciton binding energy, *E*_H_ is the energy of the 1s orbital of the H atom and m* is the effective mass), the *ε*_r_ has the potential to quantitatively evaluate photocatalytic activity.^18^ In this regard, Wang's group inserted ionic sites into the skeleton of COFs by polarization engineering, in which highly dipolar ionic groups enhance the directional polarization of COFs, resulting in ionic COFs with high *ε*_r_ relative to neutral COFs and displaying more active exciton behavior.^10^ It is evident that the *ε*_r_ is a descriptive factor to quantitatively characterize its relationship with *E*_b_ along with the photocatalytic activity which is important for the development of efficient photocatalysts.^9,10,19–22^

Pursuant to the International Roadmap for Semiconductors (ITRS), porous materials and “air gap” structures are an effective way to develop low dielectric materials.^23–25^ Porous materials are primarily designed to increase the porosity in the intrinsic structure of the material in order to effectively reduce the density of the material and thus decrease the number of polarizable dipoles in the interlayer medium.^1,10,19,26–29^ The “air gap” structure refers to where air (with a minimum *ε*_r_ of 1 for air) is mixed into a dense material , with the air eventually acting as the dielectric.^30^ Conversely, to lower the *E*_b_, the design principle of the photocatalyst entails a target along high *ε*_r_. As is known, oxygen is a radical sudden extinguisher, which greatly impedes the occurrence of controlled radical polymerizations (CRPs) and confines the industrial scalability and wide application of CRPs, and it is extremely significant to tackle this difficulty for advancing the research progress in polymer science.^31^

In the present study, we take “water-/oxygen-fueled” photo-induced electron transfer reversible addition–fragmentation chain transfer (PET-RAFT) polymerization as a demonstration platform to optimize the photocatalytic activity of COFs by narrowing the “air gap” structure of COFs through ligand tailoring, enhancing the *ε*_r_ and lowering the *E*_b_. The study confirms that the *ε*_r_ can be applied to describe the photocatalytic activity of COFs, correlates the “air gap” structure of COFs with the dielectric properties and exciton effect, and reveals the influence law of the “air gap” magnitude on the photopolymerization reaction activity.

## Results and discussion

**Fig. 1:**
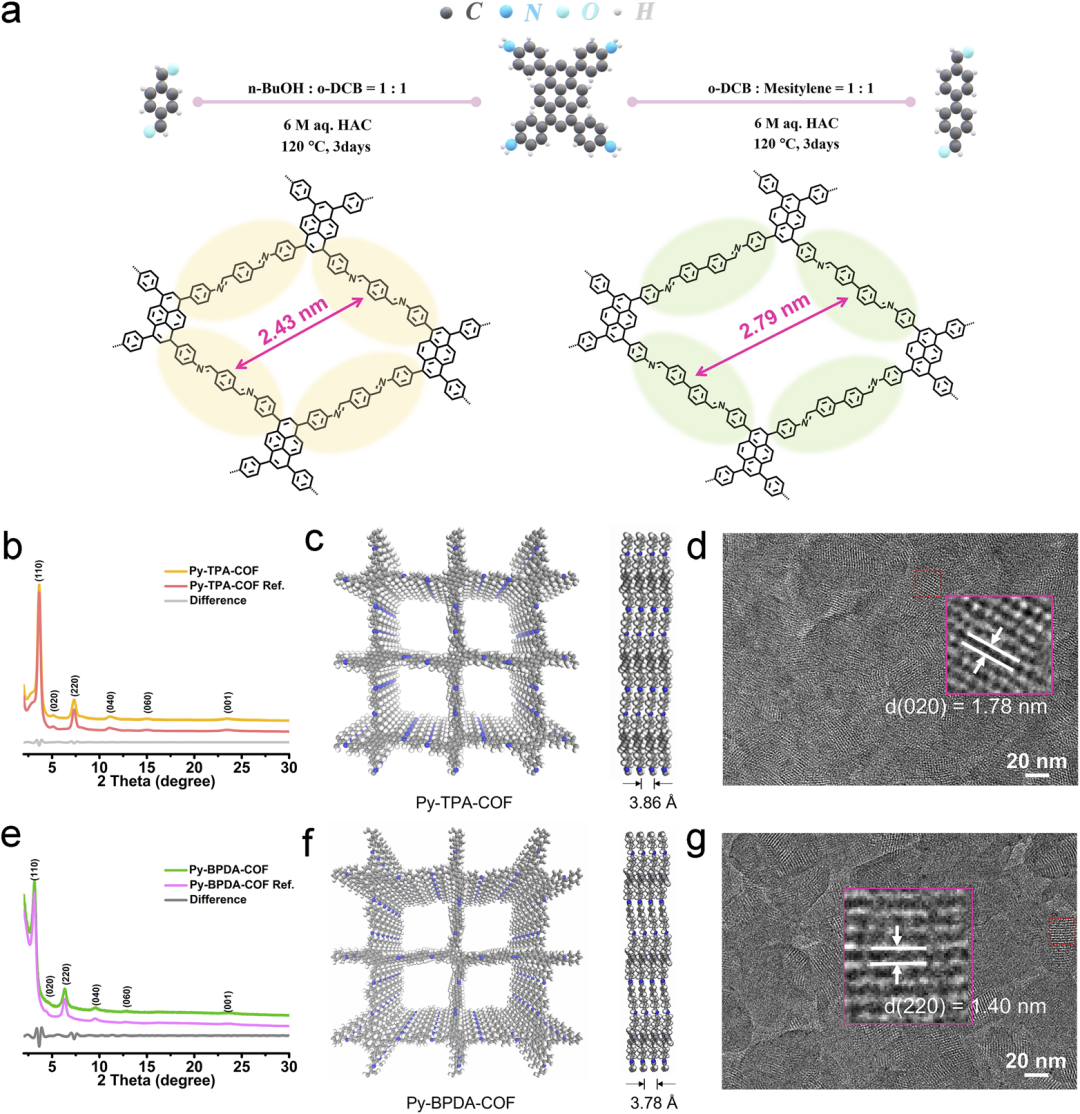
(a) Synthesis of pyrene COFs Py-TPA-COF and Py-BPDA-COF. (b) and (e) Experimental and refined P-XRD patterns of the as-prepared (b) Py-TPA-COF and (e) Py-BPDA-COF. (c) and (f) Simulated molecular structure of (c) Py-TPA-COF and (f) Py-BPDA-COF (left) with a side view (right) based on AA-stacking. (d) and (g) HR-TEM images of (d) Py-TPA-COF and (g) Py-BPDA-COF.

The crystal structures of the imine-linked Py-TPA-COF and Py-BPDA-COF were identified by powder X-ray diffraction (P-XRD) characterization incorporating theoretical structure simulations. As shown in Fig. 1b and e, the diffraction peaks at 2 theta of 3.64°, 5.10°, 7.32°, 15.03°, 23.47° and 23.61°, assigned to the (110), (020), (220), (040), (060) and (001) crystal planes of Py-TPA-COF, respectively, similarly appear at 2 theta of 3.13°, 4.40°, 6.33°, 9.52°, 12.74° and 23.61° of Py-BPDA-COF. According to the Pawley refinement results (Fig. S1†), both Py-TPA-COF and Py-BPDA-COF are matched in the *C*2/*m* space group with cell parameters of *a* = 24.0733 Å, *b* = 24.4929 Å, *c* = 3.8657 Å, *α* = 90°, *β* = 91°, and *γ* = 90° and *a* = 28.7932 Å, *b* = 27.9868 Å, *c* = 3.7906 Å, *α* = 90°, *β* = 90°, and *γ* = 90°, respectively. Meanwhile, the experimental results of Py-TPA-COF and Py-BPDA-COF are aligned highly with the results of P-XRD simulations of AA stacking, indicating that both are arranged in AA stacking mode with a layer-to-layer spacing of 3.86 Å and 3.78 Å, respectively (Fig. 1c and f). High-resolution transmission electron microscopy (HR-TEM) images (Fig. 1d and g) enable the visualization of the crystal structures, with a Py-TPA-COF lattice constant of approximately 1.78 nm, corresponding to the (020) crystal plane, and the Py-BPDA-COF lattice constant is about 1.40 nm, corresponding to its (220) crystal plane. As shown in the FT-IR spectrum (Fig. S2†), the disappearance of the –NH_2_ group of PyTTA in the vibrational band at 3341 cm^−1^ and the appearance of a peak at 1602 cm^−1^, corresponding to the C=N stretching mode of the imine, suggests the successful preparation of the target COF. ^13^C CP-MAS NMR spectra (Fig. S3†) with peaks at 160.62 ppm and 159.37 ppm illustrate the formation of imine bonds (C=N), meaning the successful assembly of the skeleton. Scanning electron microscopy (SEM) images (Fig. S4†) display Py-TPA-COF and Py-BPDA-COF as a granular morphology. The specific surface area and porosity were determined by the N_2_ adsorption–desorption method (Fig. S5†). With the help of a nonlocal density functional theory (NLDFT) model, the specific surface area of Py-TPA-COF and Py-BPDA-COF was calculated to be 1045.18 and 172.24 m^2^ g^−1^, respectively. Their corresponding pore size was 2.27 nm and 2.69 nm, respectively, which was consistent with the theoretical value, suggesting that the “air gap” of Py-TPA-COF was smaller.

**Fig. 2:**
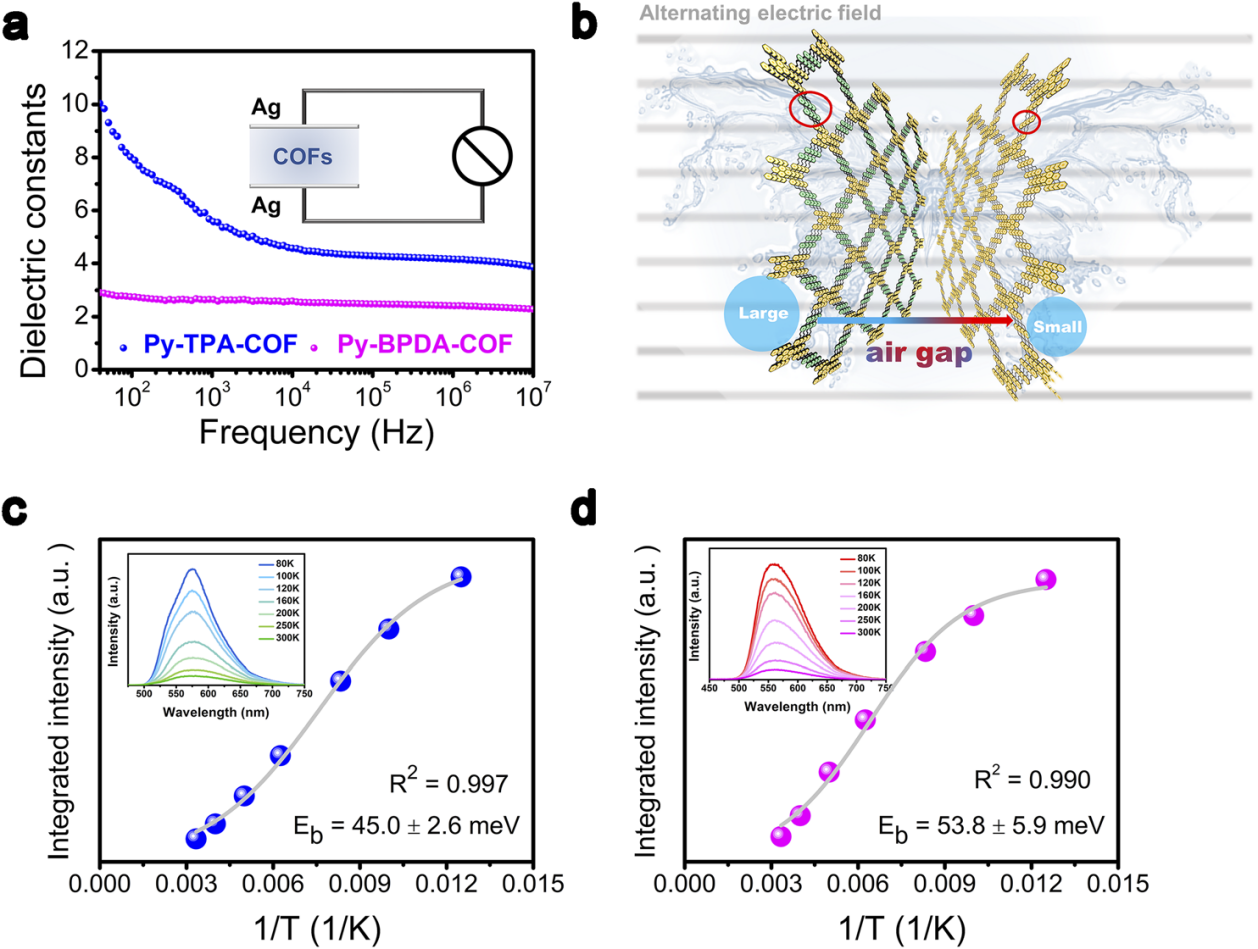
(a) Dielectric properties of Py-TPA-COF and Py-BPDA-COF dependent on frequency (inset shows a sketch of the flat plate method for measuring the dielectric constant). (b) Diagram of the “air gap” modulation of Py-TPA-COF and Py-BPDA-COF in an alternating electric field. (c) and (d) Temperature inverse *versus* Arrhenius equation for the integrated photoluminescence (PL) emission intensity of (c) Py-TPA-COF and (d) Py-BPDA-COF (illustration presents the PL spectrum *versus* temperature).

The descriptor *ε*_r_ of Py-TPA-COF and Py-BPDA-COF was measured by using the parallel plate capacitor principle on an impedance analyzer. The *ε*_r_ of Py-TPA-COF decreases with increasing frequency and outperforms the *ε*_r_ of Py-BPDA-COF in the frequency range of 40 Hz to 10 MHz (Fig. 2a). As is known, the relative *ε*_r_ of materials is frequency dependent and depends on the density of the material and the total polarizability of the molecules.^25,32^ The total polarizability can be attributed to the total contribution of electron polarization, distortion polarization or orientation polarization.^19,25,33^ Since no ionic sites are inserted in Py-TPA-COF and Py-BPDA-COF, the distortion polarization essentially does not contribute. Considering the strongly ordered skeleton of the crystalline organic polymeric material COFs and its building blocks interlock with each other through the synthesis of net chemistry, the spatial positional degrees of freedom are reduced and thus the orientation polarization is greatly suppressed.^19,20,32^ As the linker shrinks, it is possible to increase the density of the material, narrow the “air gap” structure, and raise the *ε*_r_ (Fig. 2b). To further verify that the increase in the descriptor *ε*_r_ contributes positively to the decrease in *E*_b_, temperature-dependent photoluminescence (PL) spectra were examined. As seen from the inset of Fig. 2c and d, the PL intensity of Py-TPA-COF and Py-BPDA-COF decreases upon raising the temperature. The temperature-dependent PL spectra were fitted by integration according to the Arrhenius equation (eqn (S1)†) to obtain the *E*_b_.^34^ The fitting results showed that Py-TPA-COF (*E*_b_ = 45.0 ± 2.6 meV) has a lower *E*_b_ than Py-BPDA-COF (*E*_b_ = 53.8 ± 5.9 meV), implying a relatively better charge–hole separation of Py-TPA-COF after absorbing photon energy. The above experimental results suggest that the directional reduction of the “air gap” structure by ligand modulation can lead to an increase in the descriptor *ε*_r_, which effectively reduces *E*_b_, which is consistent with the reported correlation pattern.^10^

**Fig. 3:**
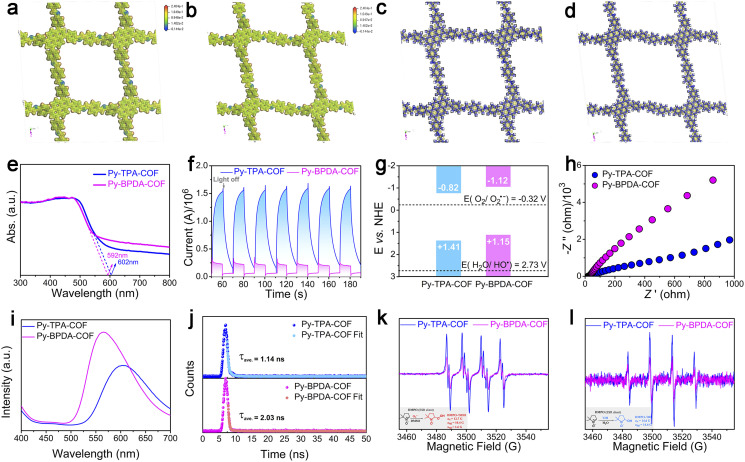
Electrostatic potential (ESP) analysis of (a) Py-TPA-COF and (b) Py-BPDA-COF at isosurface = 0.001 a.u. The charge density differences of (c) Py-TPA-COF and (d) Py-BPDA-COF at isosurface = 0.001 a.u., where blue and yellow represent the accumulation and the depletion of electrons. (e) UV-vis DRS spectra, (f) photocurrent response curves, (g) energy band location diagram, (h) electrochemical impedance spectra and (i) steady-state fluorescence spectroscopy of Py-TPA-COF and Py-BPDA-COF. (j) Time-resolved photoluminescence decay (TRPL) curves of (k) and (l) electron paramagnetic resonance (EPR) diagrams of (k) DMPO-OOH˙ adducts and (l) DMPO-OH˙ adducts of Py-TPA-COF and Py-BPDA-COF.

Density functional theory (DFT) is applied to interpret the dielectric properties and exciton dissociation behavior of the different “air gap” structures. From the electrostatic potential analysis of Py-TPA-COF and Py-BPDA-COF (Fig. 3a and b), it is seen that the positive potential is uniformly distributed over the PyTTA and linker components, while the negative potential is concentrated in the *N* atoms of the imine linker mode. The distribution conditions of positive and negative charges are similar and uniform for both, showing no local polarization. The material density of Py-TPA-COF with a short linker is larger than that of Py-BPDA-COF, which means that more dipoles can be polarized by the alternating electric field, coupled with a smaller “air gap” structure, resulting in a larger *ε*_r_ (Fig. 2b). In the difference of the ground state charge density (Fig. 3c and d), the electrons accumulate and dissipate uniformly in the skeleton, indicating again that Py-TPA-COF and Py-BPDA-COF have a uniform charge distribution.

Contraction of the “air gap” structure to raise the descriptor *ε*_r_ and thereby suppress the exciton effect enables efficient charge–hole separation and photocatalytic activity. Firstly, the light harvesting ability of Py-TPA-COF and Py-BPDA-COF was evaluated by UV-vis DRS. As seen in Fig. 3e, the absorption band edges of Py-TPA-COF and Py-BPDA-COF are 592 nm and 602 nm, respectively. The optical band gaps (*E*_g_) of Py-TPA-COF and Py-BPDA-COF were calculated to be 2.23 eV and 2.27 eV, respectively, according to the Tauc plots deduced from the Kubelka–Munk function (Fig. S6a and b†). The above results are consistent with the reported work noting the trend that the *ε*_r_ of the COF is inversely proportional to the square of the optical band gap.^9^ Subsequently, carrier transport efficiency and redox reaction potential during the photoelectrochemical conversion were studied by photoelectrochemical measurements. Transient photocurrent spectroscopy (Fig. 3f) demonstrates a stronger photocurrent signal for Py-TPA-COF than Py-BPDA-COF, signifying the more efficient charge–hole pair dissociation.^35^ The flat-band potentials (*E*_fb_) were determined by extrapolating the Mott Schottky curves to 1/*c*^2^ = 0.^36^ The *E*_fb_ of Py-TPA-COF and Py-BPDA-COF was estimated to be −0.82 and −1.12 V *vs.* NHE, respectively (Fig. S6c and d†). The bottom of the conduction band (CB) of n-type semiconductors is usually considered to be equal to the *E*_fb_.^37^ Hence, the CB potentials of Py-TPA-COF and Py-BPDA-COF are −0.82 and −1.12 V *vs.* NHE, respectively. With the *E*_g_, the valence band (VB) potentials of Py-TPA-COF and Py-BPDA-COF are +1.41 and +1.15 V *vs.* NHE, respectively (Fig. 3g). Meanwhile, its redox reaction potential was further verified by cyclic voltammetry with compatible results (Fig. S7†). Negative potentials compared to O_2_/O_2_˙^−^ (−0.33 V *vs.* NHE)^15^ suggest the feasibility of generating superoxide anions (O_2_˙^−^), and insufficiently positive potentials relative to H_2_O/OH˙ (2.73 V *vs.* NHE)^38^ make the direct oxidation of water by holes to form OH˙ an infeasible path. Electrochemical impedance analysis (Fig. 3h) shows that Py-TPA-COF possesses a smaller semicircle radius, implying a relatively small interfacial charge transfer resistance.^39^ Instantaneous photoluminescence emission spectra (Fig. 3i) at ambient temperature demonstrate the weaker charge–hole recombination efficiency of Py-TPA-COF.^38^ Meanwhile, the time-resolved photoluminescence decay (TRPL) curves (Fig. 3j) record the shorter fluorescence lifetime of Py-TPA-COF, suggesting a fast exciton dissociation behavior.^40^

Previous studies revealed that with air/oxygen as the oxidant and assisted by water, the superoxide anion was capable of rapid disproportionation to generate OH˙,^41,42^ which enabled a deep reaction. Then, the reduction capacity of photogenerated electrons of Py-TPA-COF and Py-BPDA-COF was assessed by means of EPR (Fig. 3k and l). As DMSO can suddenly inactivate OH˙ and O_2_˙^−^ are observed in DMSO.^43^ Signal peaks of DMPO-OOH˙ adducts (Fig. 3k) can be noticed in DMSO solutions, which reflect the generation of O_2_˙^−^.^44^ Among the higher DMPO-OOH˙ adduct signal peaks reveal the higher photogenerated electron reduction capacity of Py-TPA-COF leading to a relatively superior ability to generate OH˙. EPR spectra show the detection of DMPO-OH˙ adducts in H_2_O solution after illumination (Fig. 3l). The signal response intensity of DMPO-OH˙ adducts was stronger for Py-TPA-COF than for Py-BPDA-COF, indicating that more OH˙ was allowed to participate in deep reactions. In the case of H_2_O, no signal peak of DMPO-O_2_˙^−^ appeared, attributed to O_2_˙^−^ generated at the system undergoing a rapid disproportionation in aqueous solution to generate other ROS species (*e.g.*, OH˙).^45^ The production of OH˙ was again demonstrated with the assistance of the UV-vis absorption spectrum of the indicator methylene blue (MB) (Fig. S8†).

**Fig. 4:**
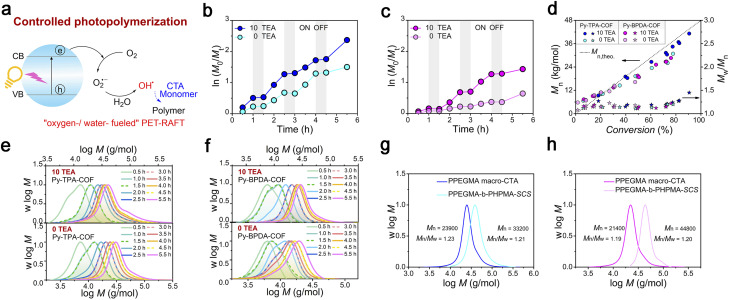
Photocatalytic activity assessment. (a) Schematic diagram of “water-/oxygen-fueled” PET-RAFT (b) and (c) kinetic plots of ln([*M*]_0_/[*M*]_*t*_) *vs.* time with different TEA concentrations of (b) Py-TPA-COF and (c) Py-BPDA-COF. (d) Evolution of *M*_n_ and *M*_w_/*M*_n_ of polymers *vs.* monomer conversion of COFs with different contents of TEA. (e) and (f) GPC normalized molecular weight distribution trace with different TEA concentrations of (e) Py-TPA-COF and (f) Py-BPDA-COF. (g) and (h) Chain extension GPC normalized molecular weight distribution trace of (g) Py-TPA-COF and (h) Py-BPDA-COF.

Photo-induced electron transfer reversible addition–fragmentation chain transfer (PET-RAFT) polymerization is mild and controllable, and is a powerful means of preparing target polymers with desired molecular weight and molecular weight distribution. The fundamental solution to the problem of oxygen blocking polymerization is to consider oxygen as the feedstock for catalytic polymerization. Therefore, “water-/oxygen-fueled” PET-RAFT polymerization was examined (Fig. 4a). As shown in the polymerization kinetic curves (Fig. 4b, c, Tables S1 and 3†), Py-TPA-COF exhibits superior catalytic performance under the same experimental conditions with or without TEA addition. The obtained polymers all maintain a low molecular weight distribution (*M*_w_/*M*_n_ < 1.5) and have molecular weight (*M*_n_) close to the theoretical line (Fig. 4d). Simultaneously, the *M*_n_, *M*_w_/*M*_n_ and GPC elution trace (Fig. 4e and f) of the polymer almost overlap at each intermittent light start-stop operation revealing its high light dependence. Notably, the GPC curve shows a shoulder peak early in the polymerization (Fig. 4e and f), which is caused by the presence of multiple starting radicals at the initiation of polymerization. As seen from the LC-QTOF-MS spectral analysis (Fig. S12†), the peak distributions of multiple sequences are spaced at the exact mass of the HPMA repeat unit (144.08 g mol^−1^). The polymerization eventually goes into the RAFT cycle with increasing light time. *In situ* chain expansion experiments (Fig. 4g, h and S13†) demonstrate the high activity of the polymer chains, with the GPC curve shifting in the direction of increasing molecular weight with the addition of the second segment monomer, implying the retention of the thiocarbonyl sulfide group (–SCS–).

**Fig. 5:**
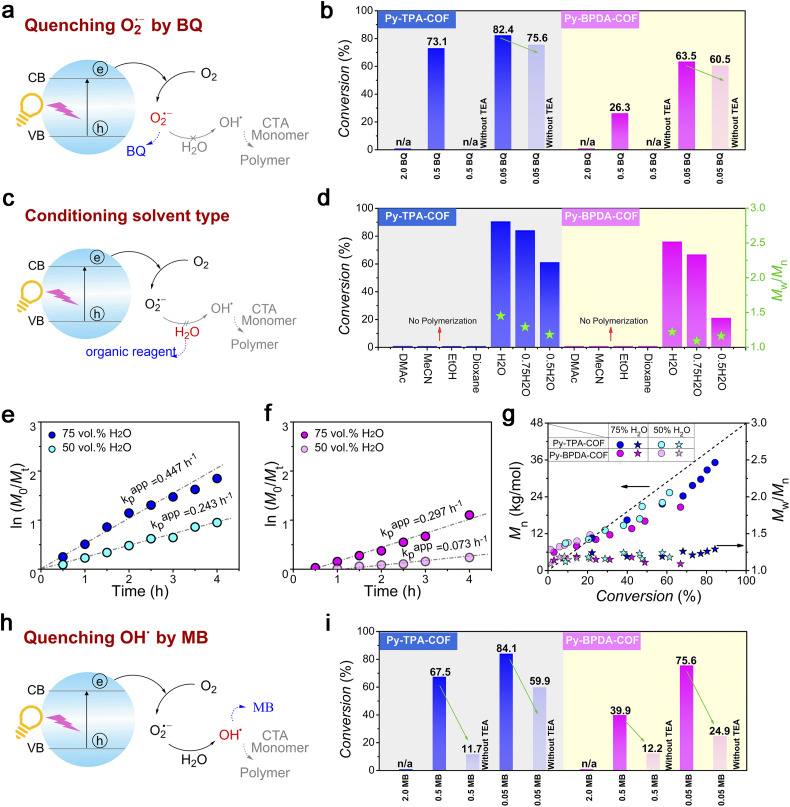
Evidence for controlled experiments. (a) Schematic diagram of O_2_˙^−^ removal by BQ. (b) Final polymer conversion *vs.* BQ concentrations with COFs. (c) Schematic diagram of organic reagent substitution for H_2_O. (d) Final polymer conversion and *M*_w_/*M*_n_*vs.* solvent types with COFs. (e) and (f) Kinetic plots of ln([*M*]_0_/[*M*]_*t*_) *vs.* time with different TEA concentrations of (e) Py-TPA-COF and (f) Py-BPDA-COF. (g) Evolution of *M*_n_ and *M*_w_/*M*_n_ of polymers *vs.* monomer conversions of COFs with different contents of H_2_O. (h) Schematic diagram of OH˙ removal by MB. (i) Final polymer conversions *vs.* MB concentration with COFs.

To dissect the photocatalytic reaction mechanism of Py-TPA-COF and Py-BPDA-COF, the main variables were modulated. When the O_2_˙^−^ anion was quenched by a sufficient amount of BQ, the polymerization process did not occur in either case and was alleviated as the amount of BQ decreased (Fig. 5a, b, Tables S5 and 6†). The absence of O_2_˙^−^ inevitably hinders the generation of OH˙ thereby inhibiting the polymerization reaction. Similarly, when another fuel water was neglected, the pathway of OH˙ generation was hindered (Fig. 5c). Control experiments (Table S2 entries 3–6 and Table S4 entries 3–6†) demonstrated that the polymerization process does not occur in non-aqueous solvents (DMAc/MeCN/EtOH/dioxane), due to the absence of water obstructing the pathway of OH˙ generation. Furthermore, it was found that the polymerization rate increased as the water content of the system increased in the compounding experiments of the good solvent water with the bad solvent dioxane, which further verified the above results (Fig. 5e–g; S8–10; Table S2 entries 1 and 2 and Table S4 entries 1 and 2†). Not surprisingly, the polymerization process did not occur when the OH˙ were scavenged by a sufficient amount of MB, and by diluting the amount of MB, the generation of OH˙ was allowed to initiate polymerization (Fig. 5h and i). The presence of oxygen limits the industrial scalability and wide application of controlled radical polymerization, whereas the strong oxidative properties of OH˙ enable controlled radical polymerization in an open subsurface environment. The feasibility of polymerization processing in an open environment provides an interesting strategy for advancing research in polymer science. Finally, we evaluated the cycling stability of Py-TPA-COF and Py-BPDA-COF (Fig. S14†). The chemical stability of Py-TPA-COF and Py-BPDA-COF was illustrated by XRD as well as FT-IR after 24 h immersion treatment under reaction system conditions. Py-TPA-COF and Py-BPDA-COF maintained high activity after five experimental cycles of photopolymerization, and the slight decrease in activity could be attributed to the decrease in crystallinity.

To further evidence that the *ε*_r_ is a promising descriptor of *E*_b_, an additional pair of TFP-COFs with different air gap structures were investigated by considering the pore size as a single factor. Analogously, TFP-1Ben with smaller air gap structures has higher *ε*_r_, lower *E*_b_ and more efficient high catalytic activity relative to TFP-2Ben (Fig. S15–19†). Experimental results suggest that COFs with smaller pore size have better photocatalytic performance when the air gap structure is regarded as a single influencing factor, revealing that the *ε*_r_ descriptor has the potential to facilitate reverse on-demand photocatalyst design, which will provide a new strategy for the design and development of more efficient photocatalytic systems.

## Conclusions

In summary, this work proposes an experimentally measurable *ε*_r_ parameter as a descriptor to quantitatively describe its relationship with *E*_b_ and photocatalytic activity. By shortening the ligand to target the “air gap” structure of the COF, the descriptor *ε*_r_ is increased to lower the *E*_b_ and promote the dissociation of excitons to form free carriers to obtain higher catalytic activity. At the same time, the RAFT polymerization protocol using water and oxygen as fuel supply is explored in an open environment, which is important for the development of polymer science and the evolution of efficient photocatalysis.

## Data availability

All relevant data supporting this article have been included in the main text and the ESI.† All original data generated during this work are available from the corresponding authors upon request.

## Author contributions

H. Y. proposed the research idea, preparation and characterization of the catalyst and wrote the manuscript. Z. L. explored the synthesis method of the catalyst. X. Y. proposed the test method of dielectric properties and the revision of the manuscript. S. W. proposed the idea of dielectric property testing. L. H. contributed to the revision of the manuscript and the financial sponsorship.

## Conflicts of interest

There are no conflicts to declare.

## Supplementary Material

SC-014-D3SC01719B-s001
