# Supplementary material for: Influence laws of air gap structure manipulation of covalent organic frameworks on dielectric properties and exciton effects for photopolymerization
Source: Chem Sci. 2023 Jul 5;14(30):8095–102. doi: 10.1039/d3sc01719b (PMC10395304; doi:10.1039/d3sc01719b)
Supplement: SC-014-D3SC01719B-s001 [file SC-014-D3SC01719B-s001.pdf]

Electronic Supplementary Material (ESI) for Chemical Science.
This journal is © The Royal Society of Chemistry 2023

Supplemental information

Influence Laws of Air Gap Structure Manipulation of Covalent Organic Frameworks on Dielectric Properties and Exciton Effects for Photopolymerization

Hongjie Yang, Zhen Lu, Xiangyu Yin, Shengjin Wu, Linxi Hou*

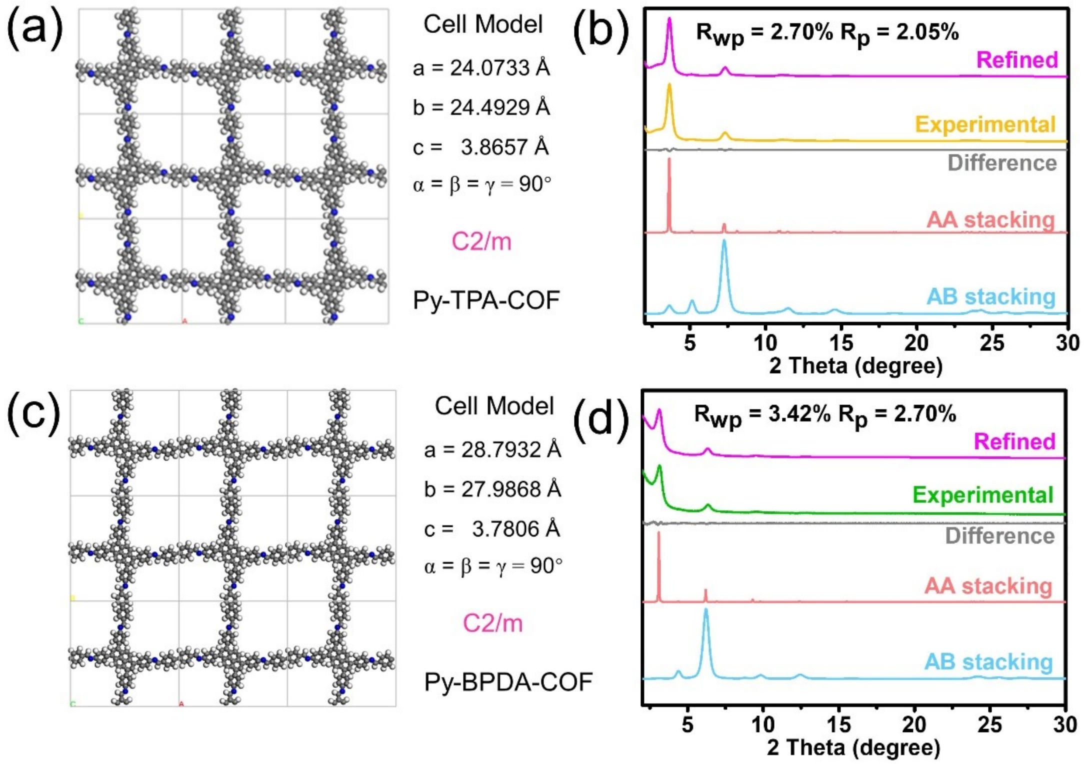

Figure S1. Simulated molecular structure of (a) Py-TPA-COF and (c) Py-BPDA-COF. (b) and (d) P-XRD patterns of (b) Py-TPA-COF and (d) Py-BPDA-COF with the refined profiles, experimental profiles, difference, simulated AA/AB stacking model.

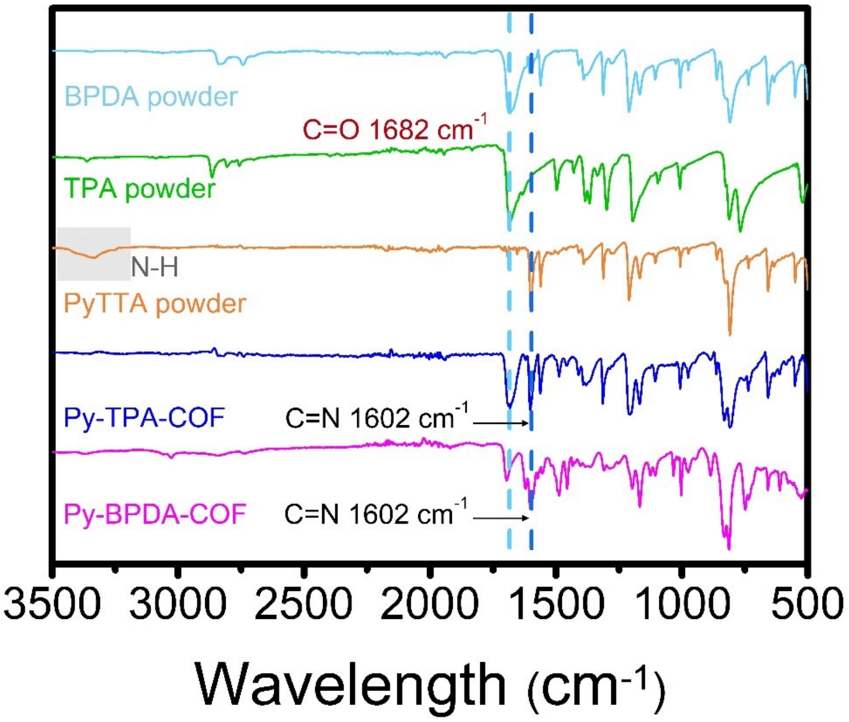

Figure S2. FT-IR spectra of Py-TPA-COF, Py-BPDA-COF, PyTTA, TPA and BPDA.

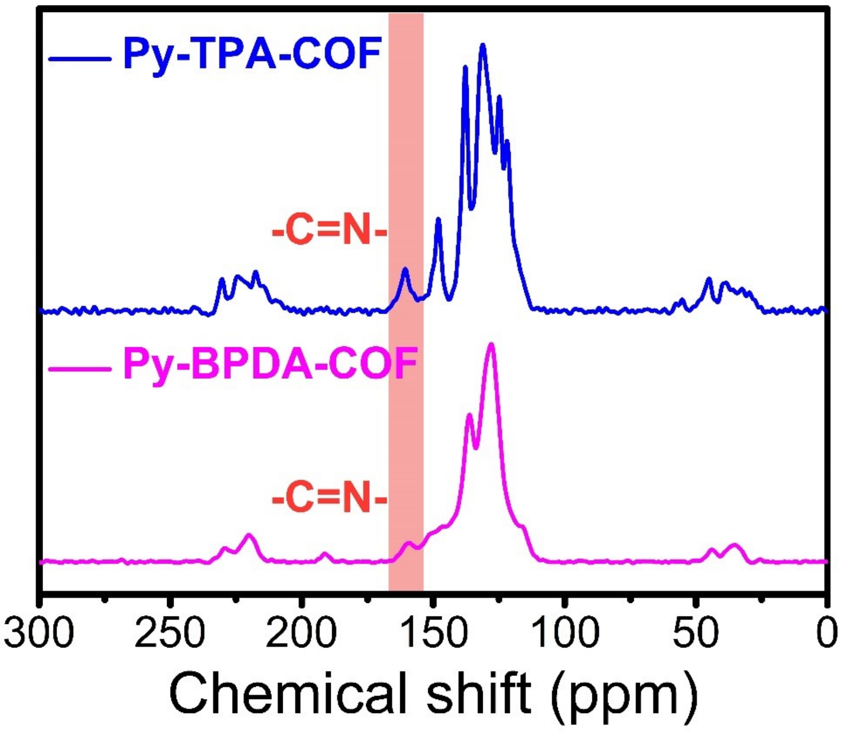

Figure S3. ^{13}C CP-MAS solid state NMR spectrum of Py-TPA-COF and Py-BPDA-COF.

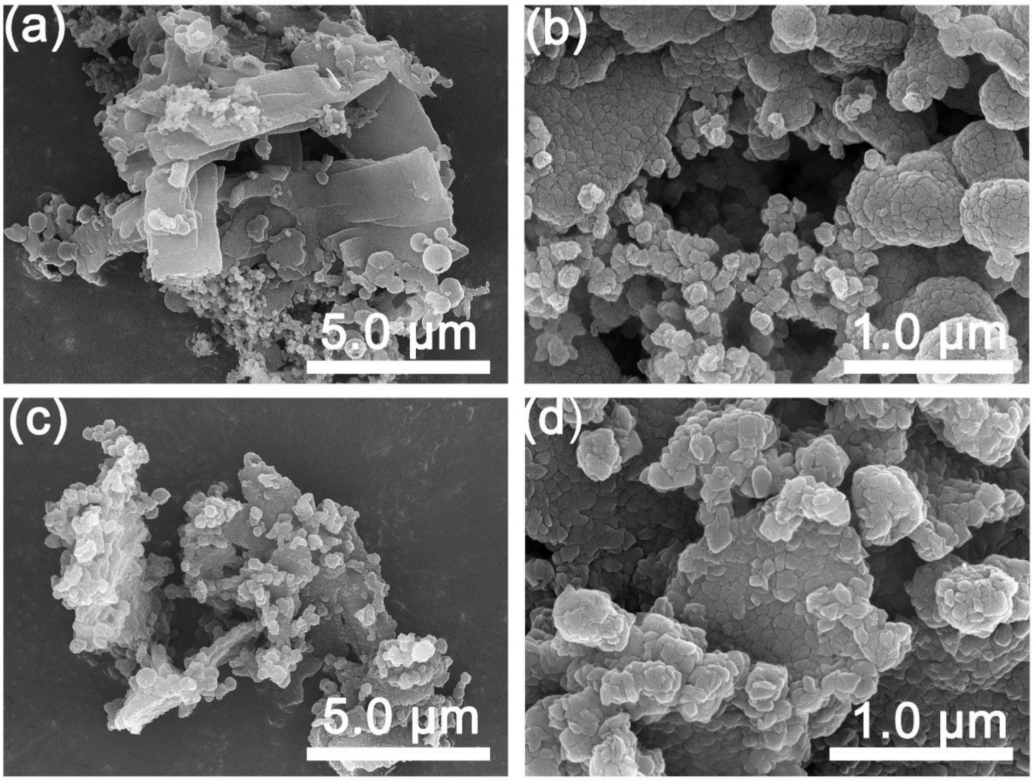

Figure S4. SEM images of Py-TPA-COF with (a) 5.0 μm and (b) 1.0 μm.

SEM images of Py-BPDA-COF with (c) 5.0 μm and (d) 1.0 μm.

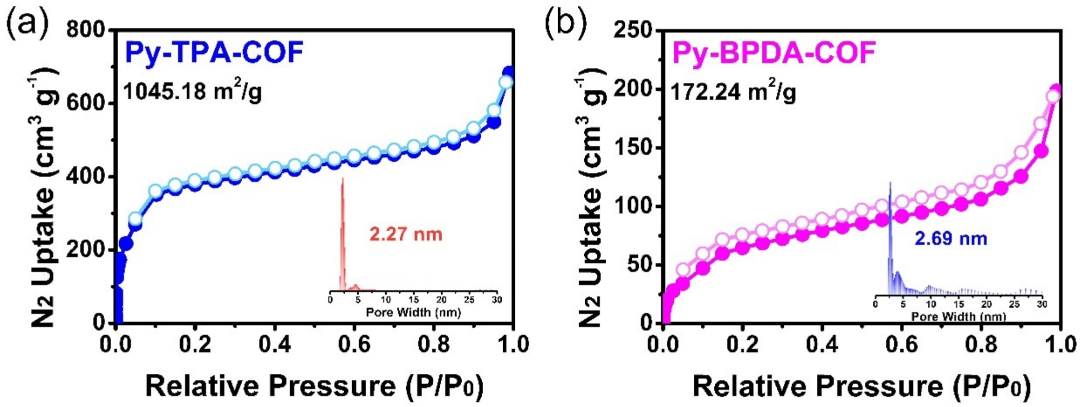

Figure S5. Nitrogen isothermal adsorption and desorption curves of (a) Py-TPA-COF and (b) Py-BPDA-COF.

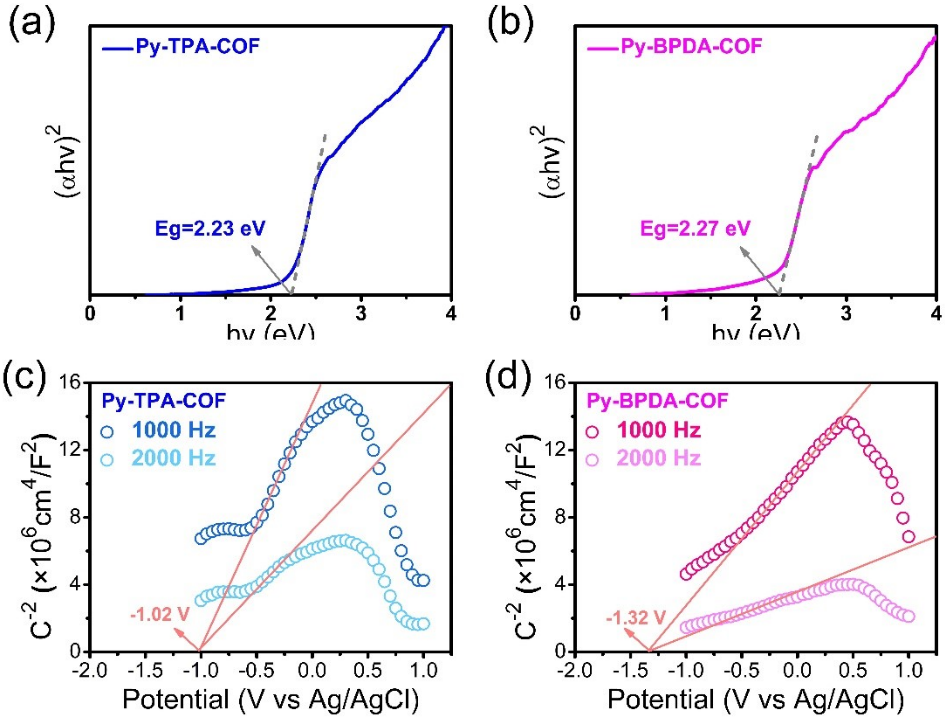

Figure S6. Tauc plots obtained with Kubelka-Munk functions and linear fits for optical band gaps of (a) Py-TPA-COF and (b) Py-BPDA-COF. Mott-Schottky plots with 1000 Hz and 2000 Hz of (c) Py-TPA-COF and (d) Py-BPDA-COF.

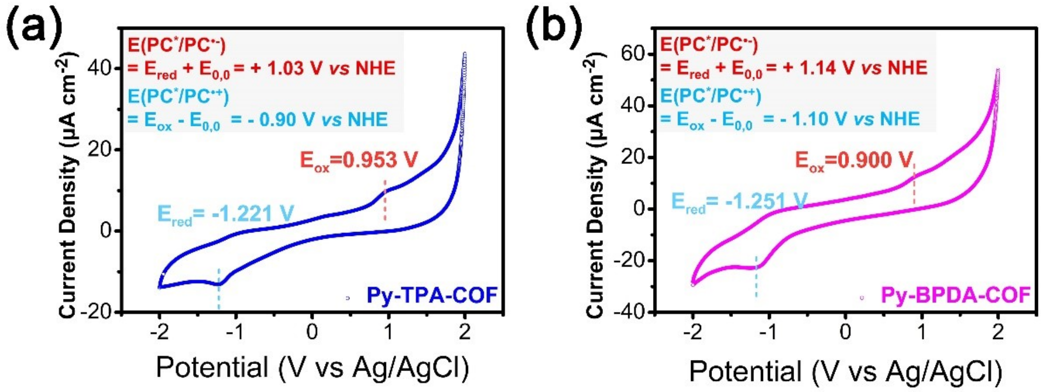

Figure S7. Cyclic voltametric curves of (a) Py-TPA-COF and (b) Py-BPDA-COF.

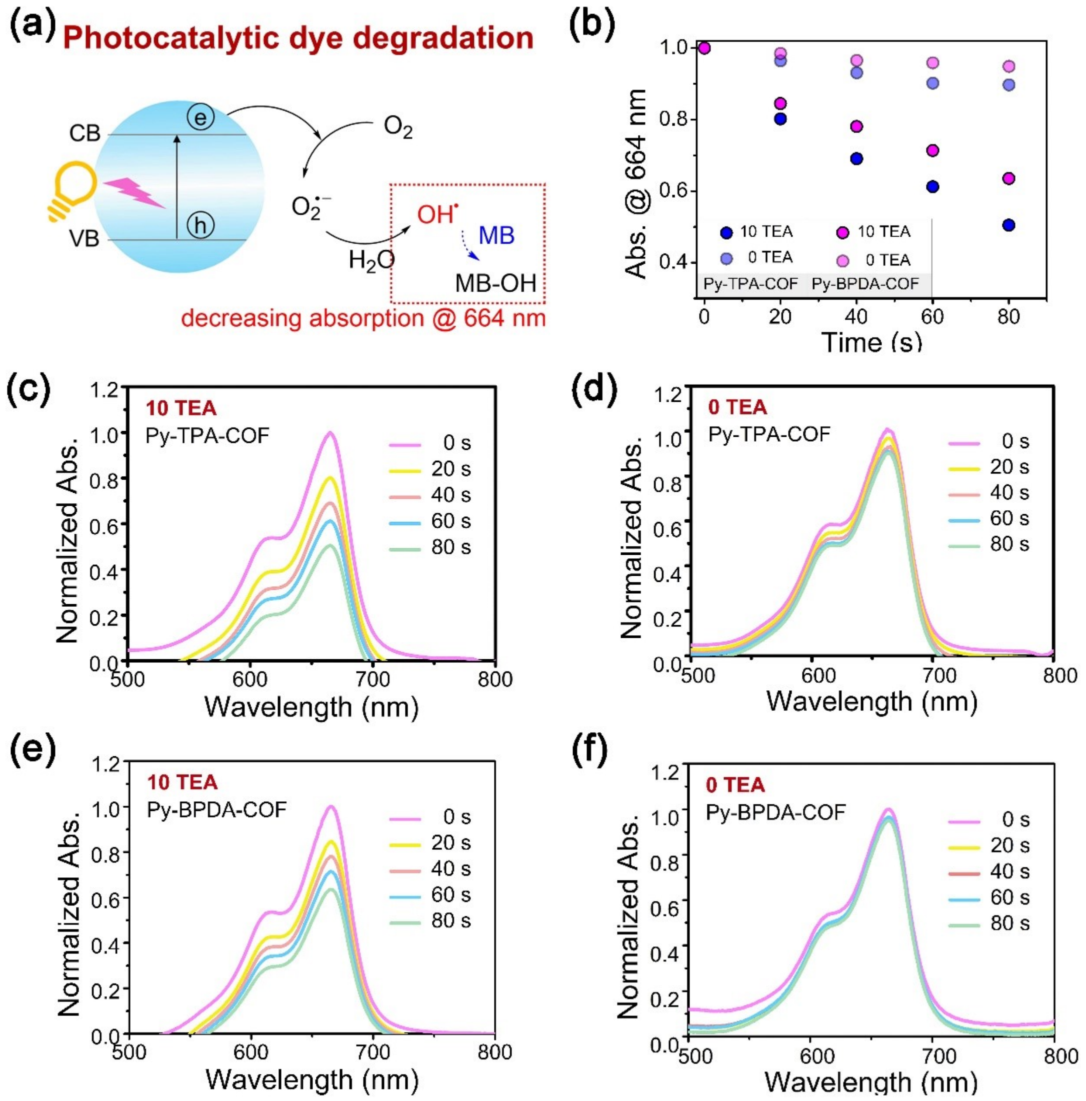

Figure S8. (a) Illustration of UV-vis absorption of MB. (b) Time-resolved UV-vis absorption of MB at 664 nm. (c) and (d) UV-vis absorption spectra of MB with Py-BPDA-COF at (c) 10 eq. TEA and (d) 0 eq. TEA. (e) and (f) UV-vis absorption spectra of MB with Py-BPDA-COF at (e) 10 eq. TEA and (f) 0 eq. TEA.

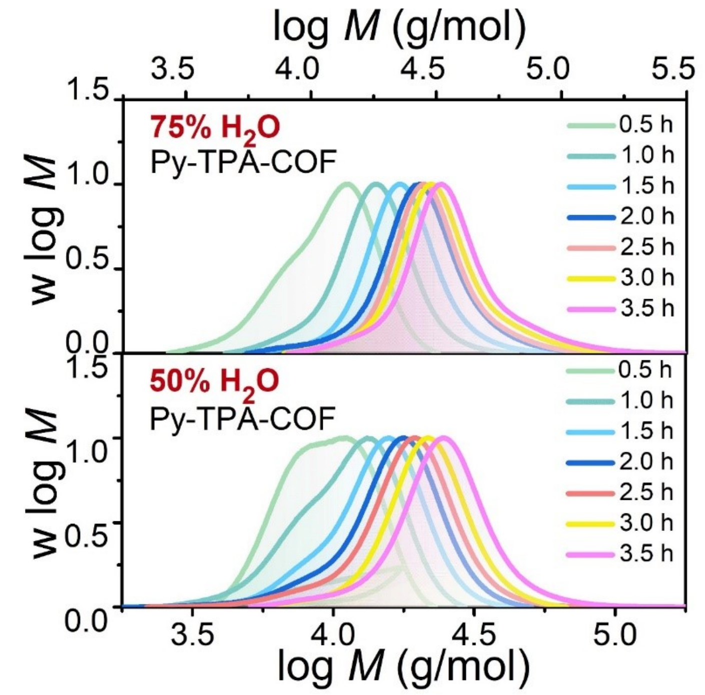

Figure S9. GPC normalized molecular weight distribution trace of varying different concentration of different volume of H_2O with Py-TPA COF.

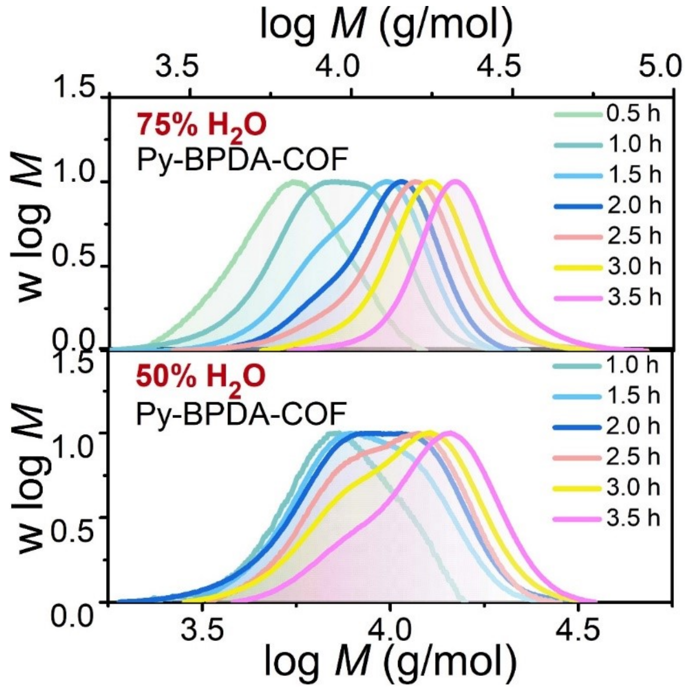

Figure S10. GPC normalized molecular weight distribution trace of varying different concentration of different volume of H_2O with Py-BPDA COF.

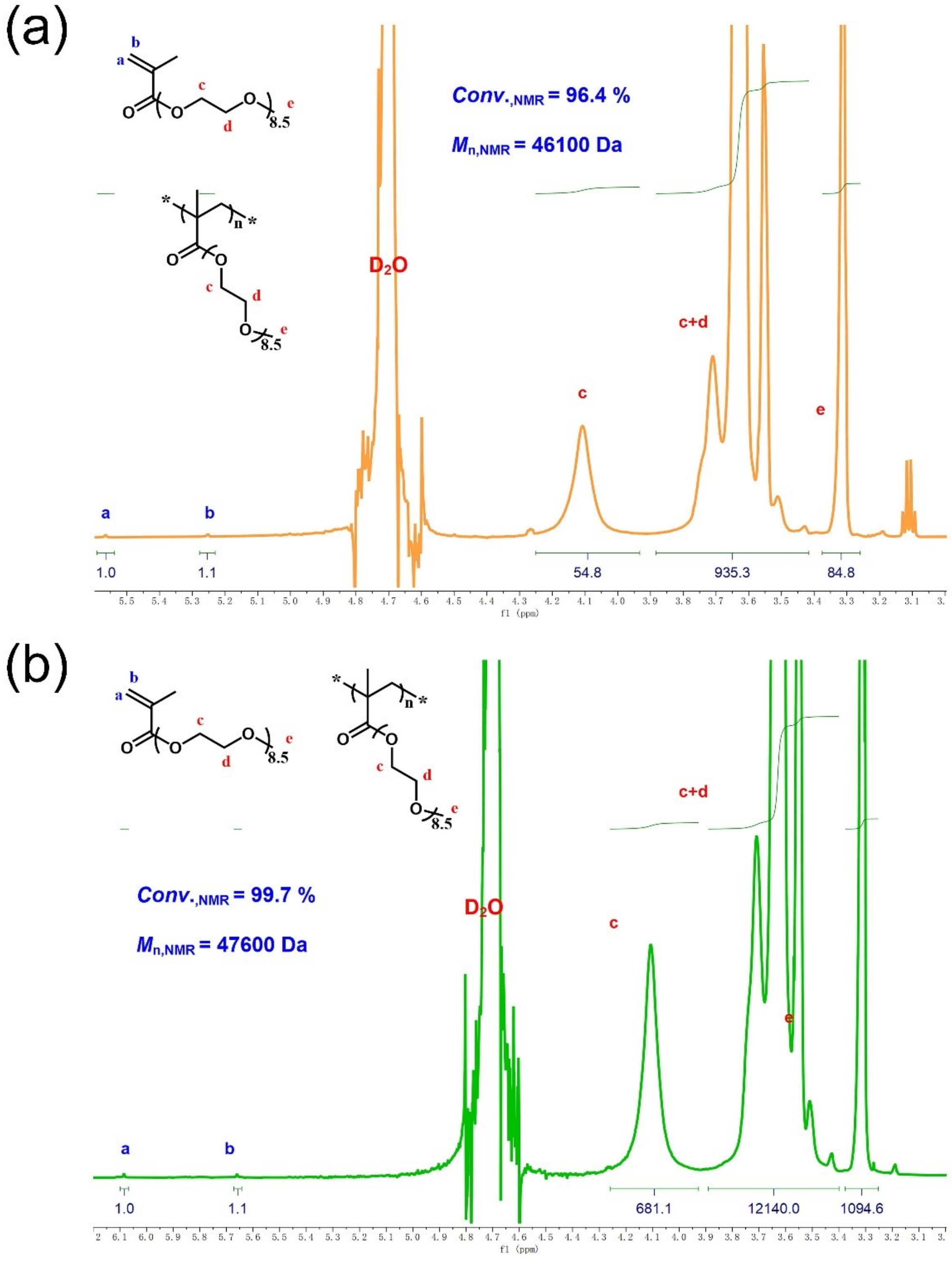

Figure S11. ^{1}H NMR spectra of obtained crude product catalyzed by (a) Py-TPA-COF and (b) Py-BPDA-COF in D_2O ([$PEGMA_{475}$]: [CPADB]: [TEA]= 100: 1: 10, with 10 mg catalyst).

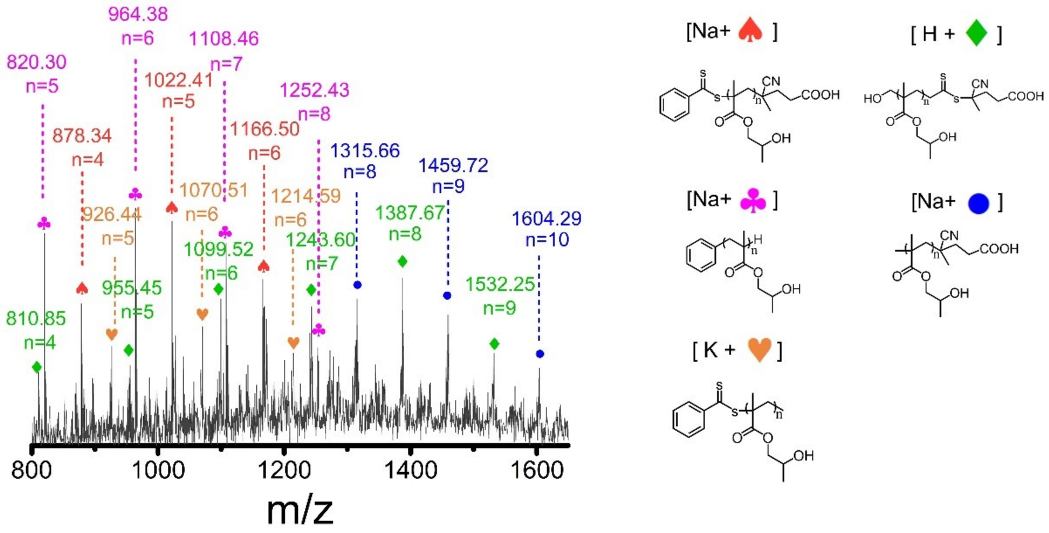

Figure S12. LC-QTOF-MS spectrometry with HPMA (target degrees of polymerization, DP = 20).

(a)

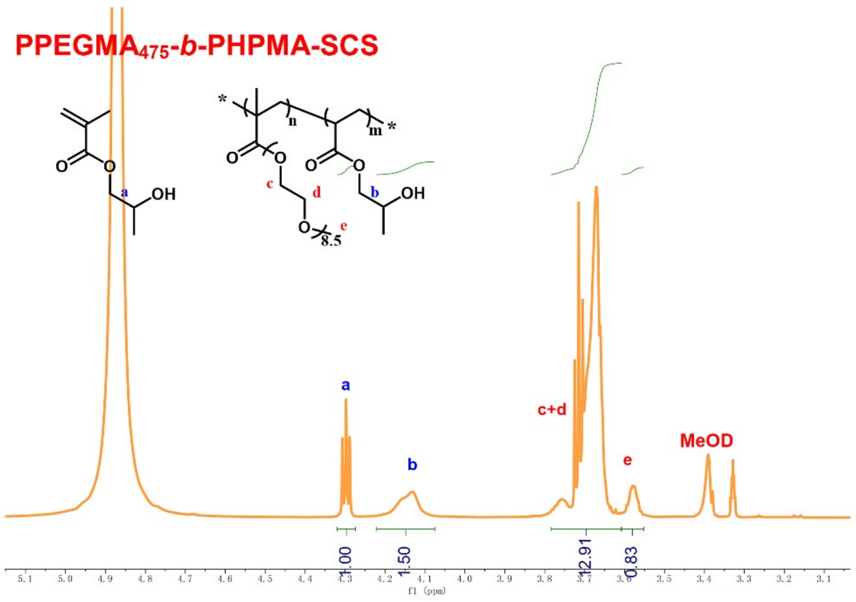

(b)

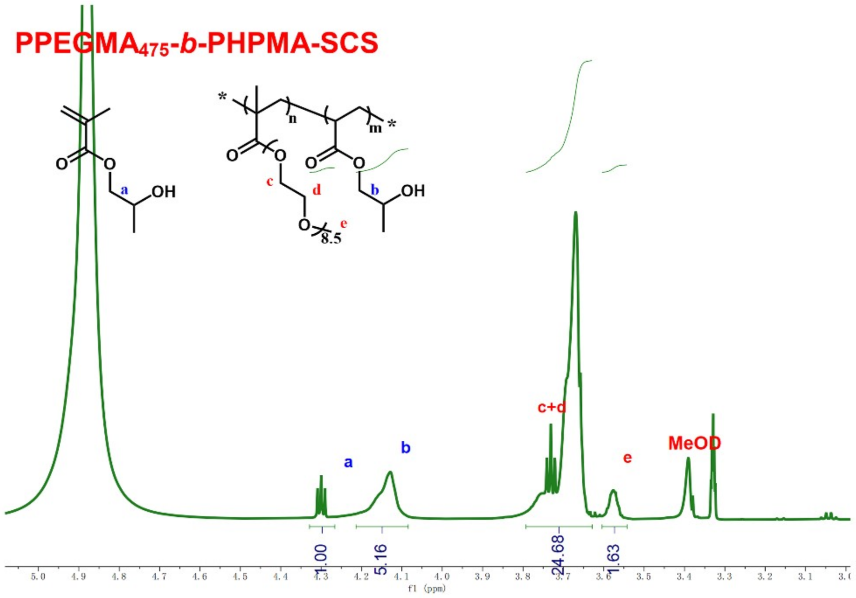

Figure S13. Chain extension experiments. ^{1}H NMR spectra of obtained crude product PPEGMA$_{475}$-*b*-PHPMA-*SCS* catalyzed by (a) Py-TPA-COF and (b) Py-BPDA-COF in D_2O (targeted DP = 150, with 10 eq. TEA).

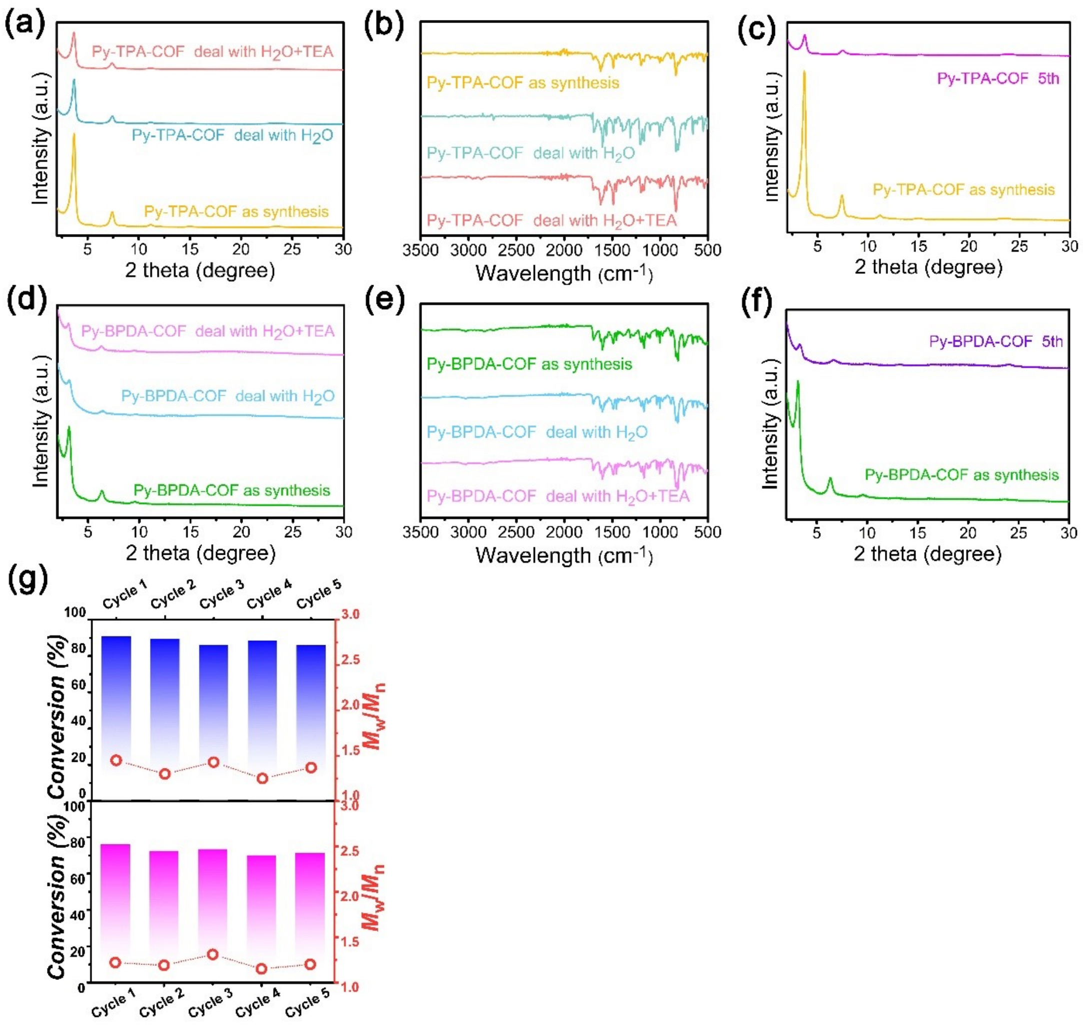

Figure S14. (a) and (d) XRD patterns and (b) and (e) FT-IR spectra of (a) and (b) Py-TPA-COF and (d) and (e) Py-BPDA-COF upon 1-day treatment in different conditions. (b) XRD patterns after 5th cycle of Py-TPA-COF and (f) Py-BPDA-COF. (g). Recycle experiment under air condition. The bars in the graph indicate the final conversion (%) of monomer, while the pink circles indicate the M_w/M_n of the final polymer.

Table S1. Results of "oxygen- /water- fueled" PET-RAFT polymerization with Py-TPA-COF.

#[a]	COF	$[PEGMA_{475}]$: [CPADB]: [TEA]	T (h)	$M_{n,GPC}$[b] (kg/mol)	$M_{n,th}$[c] (kg/mol)	α[d] (%)	M_w/M_n[b]
1	Py-TPA-COF.	100:1:10	0.5	9.3	8.9	18.2	1.27
			1	19.0	19.2	39.7	1.15
			1.5	18.3	19.9	41.3	1.19
			2	26.7	29.0	60.5	1.19
			2.5	31.3	34.5	72.0	1.19
			3	30.4	34.9	72.9	1.21
			3.5	34.5	37.0	77.4	1.24
			4	36.8	39.3	82.1	1.27
			4.5	36.3	39.6	82.7	1.28
			5	41.2	43.3	90.7	1.45
2	Py-TPA-COF.	100:1:0	0.5	6.6	3.2	6.1	1.12
			1	10.1	10.0	20.4	1.15
			1.5	10.2	10.5	21.6	1.14
			2	14.5	17.0	35.2	1.14
			2.5	19.1	23.0	47.8	1.12
			3	19.3	23.6	49.1	1.11
			3.5	23.5	29.1	60.7	1.13
			4	26.8	34.7	72.5	1.19
			4.5	26.1	35.1	73.4	1.20
			5	30.8	37.2	77.7	1.20

[a] Reaction was performed at room temperature with white LED irradiation (13 W m^{-1}, 15 mW cm^{-2}) with 10 mg Py-TPA-COF. [b] Molecular weight and molecular weight distribution (M_w/M_n) were determined by GPC analysis (THF as eluent) calibrated with PMMA standards. [c] $M_{n,th}$ = MW (initiator) + MW (monomer) × Conv. × ([monomer]/[initiator]), where MW means molecular weight. [d] Monomer conversion was determined by ^1H NMR (Refer to Figure S11).

Table S2. Results of the effect on the reaction solvent for "oxygen- /water-fueled" PET-RAFT polymerization with Py-TPA-COF.

#[a]	Solvent	T(h)	$M_{n,GPC}$[b](kg/mol)	$M_{n,th}$[c](kg/mol)	α[d](%)	M_w/M_n[b]
1	H_2O/Dioxane (75%/25%)	0.5	10.6	10.8	22.2	1.24
		1	16.4	19.2	39.9	1.19
		1.5	21.7	27.7	57.6	1.19
		2	24.3	32.6	68.1	1.26
		2.5	27.7	34.9	72.9	1.21
		3	29.7	36.9	77.1	1.23
		3.5	32.7	38.4	80.3	1.26
		4	35.2	40.3	84.3	1.29
2	H_2O/Dioxane (50%/50%)	0.5	8.9	4.5	8.8	1.12
		1	10.2	10.0	20.5	1.18
		1.5	13.1	13.9	28.8	1.16
		2	14.7	18.4	38.1	1.24
		2.5	16.8	22.3	46.3	1.23
		3	20.8	22.9	47.5	1.16
		3.5	22.1	27.7	57.7	1.24
		4	25.4	29.4	61.3	1.18
3	Dioxane	16.0	-	-	-	-
4	DMAc	16.0	-	-	-	-
5	MeCN	16.0	-	-	-	-
6	EtOH	16.0	-	-	-	-

[a] Reaction was performed at room temperature with white LED irradiation (13 W m^{-1}, 15 mW cm^{-2}) with 10 mg Py-TPA-COF under the ratio of [$PEGMA_{475}$]: [CPADB]: [TEA] = 100: 1: 10. [b] Molecular weight and molecular weight distribution (M_w/M_n) were determined by GPC analysis (THF as eluent) calibrated with PMMA standards. [c] $M_{n,th}$ = MW (initiator) + MW (monomer) × Conv. × ([monomer]/[initiator]), where MW means molecular weight. [d] Monomer conversion was determined by ^1H NMR (Refer to Figure S11).

Table S3. Results of “oxygen- /water- fueled” PET-RAFT polymerization with Py-BPDA-COF.

#[a]	COF	[$PEGMA_{475}$]: [CPADB]: [TEA]	T (h)	$M_{n,GPC}$[b] (kg/mol)	$M_{n,th}$[c] (kg/mol)	α[d] (%)	M_w/M_n[b]
1	Py-BPDA-COF.	100:1:10	0.5	7.3	3.3	6.5	1.16
			1	9.0	7.3	14.9	1.19
			1.5	9.1	6.7	13.6	1.15
			2	13.5	14.6	30.2	1.15
			2.5	19.5	23.8	49.5	1.11
			3	18.8	24.3	50.5	1.15
			3.5	25.2	30.8	64.2	1.12
			4	26.7	34.4	71.8	1.15
			4.5	26.6	34.6	72.3	1.15
			5	28.6	36.4	76.1	1.22
2	Py-BPDA-COF.	100:1:0	0.5	5.8	1.1	1.7	1.08
			1	7.1	4.0	7.9	1.12
			1.5	6.8	4.2	8.3	1.17
			2	9.5	7.3	14.8	1.15
			2.5	10.9	9.8	20.0	1.15
			3	10.9	9.9	20.2	1.15
			3.5	11.5	13.1	26.9	1.23
			4	13.9	15.0	31.0	1.18
			4.5	14.4	15.1	31.1	1.14
			5	16.3	22.8	47.5	1.18

[a] Reaction was performed at room temperature with white LED irradiation (13 W m^{-1}, 15 mW cm^{-2}) with 10 mg Py-BPDA-COF. [b] Molecular weight and molecular weight distribution (M_w/M_n) were determined by GPC analysis (THF as eluent) calibrated with PMMA standards. [c] $M_{n,th}$ = MW (initiator) + MW (monomer) × Conv. × ([monomer]/[initiator]), where MW means molecular weight. [d] Monomer conversion was determined by ^1H NMR (Refer to Figure S11).

Table S4. Results of the effect on the reaction solvent for "oxygen- /water-fueled" PET-RAFT polymerization with Py-BPDA-COF.

#[a]	Solvent	T(h)	$M_{n,GPC}$[b](kg/mol)	$M_{n,th}$[c](kg/mol)	α[d](%)	M_w/M_n[b]
1	H_2O/Dioxane (75%/25%)	0.5	6.0	1.7	3.0	1.14
		1	8.0	7.1	14.4	1.19
		1.5	10.1	11.8	24.2	1.15
		2	11.6	15.1	31.2	1.15
		2.5	13.7	20.3	42.3	1.18
		3	16.1	23.5	48.9	1.11
		3.5	20.7	32.0	66.9	1.09
		4	6.0	1.7	3.0	1.14
2	H_2O/Dioxane (50%/50%)	0.5	-	-	-	-
		1	6.8	0.9	1.3	1.09
		1.5	7.8	3.0	5.8	1.18
		2	8.2	4.2	8.3	1.19
		2.5	9.1	5.1	10.2	1.15
		3	9.6	7.2	14.6	1.17
		3.5	11.6	10.4	21.3	1.16
		4	6.8	0.9	1.3	1.09
3	Dioxane	16.0	-	-	-	-
4	DMAc	16.0	-	-	-	-
5	MeCN	16.0	-	-	-	-
6	EtOH	16.0	-	-	-	-

[a] Reaction was performed at room temperature with white LED irradiation (13 W m^{-1}, 15 mW cm^{-2}) with 10 mg Py-BPDA-COF under the ratio of [$PEGMA_{475}$]: [CPADB]: [TEA] = 100: 1: 10. [b] Molecular weight and molecular weight distribution (M_w/M_n) were determined by GPC analysis (THF as eluent) calibrated with PMMA standards. [c] $M_{n,th}$ = MW (initiator) + MW (monomer) × Conv. × ([monomer]/[initiator]), where MW means molecular weight. [d] Monomer conversion was determined by ^{1}H NMR (Refer to Figure S11).

Table S5. Results of the "oxygen- /water- fueled" PET-RAFT polymerization with radical scavenger using Py-TPA-COF as photocatalyst under full air condition in aqueous.

#[a]	Quencher	Role	$[PEGMA_{475}]$: [CPADB]: [TEA]: [Quencher]	T (h)	$M_{n,GPC}$[b] (kg/mol)	α[c] (%)	M_w/M_n[b]
1	MB	$OH^{\bullet}$ scavenger	100:1:10:0.5	4	25.7	73.1	1.10
2	MB	$OH^{\bullet}$ scavenger	100:1:0:0.5	4	-	-	-
3	MB	$OH^{\bullet}$ scavenger	100:1:10:0.05	4	29.7	82.4	1.12
4	MB	$OH^{\bullet}$ scavenger	100:1:0:0.05	4	28.6	75.6	1.13
5	MB	$OH^{\bullet}$ scavenger	100:1:10:2	4	-	-	-
6	BQ	$OH^{\bullet}$ scavenger	100:1:10:0.5	4	25.2	67.5	1.11
7	BQ	$O_2^{\bullet -}$ scavenger	100:1:0:0.5	4	7.2	11.7	1.18
8	BQ	$O_2^{\bullet -}$ scavenger	100:1:10:0.05	4	22.5	84.1	1.11
9	BQ	$O_2^{\bullet -}$ scavenger	100:1:0:0.05	4	21.3	59.9	1.10
10	BQ	$O_2^{\bullet -}$ scavenger	100:1:10:2	4	-	-	-

[a] Reaction was performed at room temperature with white LED irradiation (13 W m^{-1}, 15 mW cm^{-2}) with 10 mg Py-TPA-COF. [b] Molecular weight and molecular weight distribution (M_w/M_n) were determined by GPC analysis (THF as eluent) calibrated with PMMA standards. [c] $M_{n,th}$ = MW (initiator) + MW (monomer) × Conv. × ([monomer]/[initiator]), where MW means molecular weight. [d] Monomer conversion was determined by ^1H NMR (Refer to Figure S11).

Table S6. Results of the "oxygen- /water- fueled" PET-RAFT polymerization with radical scavenger using Py-BPDA-COF as photocatalyst under full air condition in aqueous.

#[a]	Quencher	Role	$[PEGMA_{475}]$: [CPADB]: [TEA]: [Quencher]	T (h)	$M_{n,GPC}$[b] (kg/mol)	α[c] (%)	M_w/M_n[b]
1	MB	$OH^{\bullet}$ scavenger	100:1:10:0.5	4	13.6	26.3	1.14
2	MB	$OH^{\bullet}$ scavenger	100:1:0:0.5	4	-	-	-
3	MB	$OH^{\bullet}$ scavenger	100:1:10:0.05	4	24.7	63.5	1.11
4	MB	$OH^{\bullet}$ scavenger	100:1:0:0.05	4	19.7	60.6	1.15
5	MB	$OH^{\bullet}$ scavenger	100:1:10:2	4	-	-	-
6	BQ	$OH^{\bullet}$ scavenger	100:1:10:0.5	4	18.6	39.9	1.11
7	BQ	$O_2^{\bullet-}$ scavenger	100:1:0:0.5	4	8.1	12.2	1.16
8	BQ	$O_2^{\bullet-}$ scavenger	100:1:10:0.05	4	31.5	75.6	1.15
9	BQ	$O_2^{\bullet-}$ scavenger	100:1:0:0.05	4	12.5	24.9	1.12
10	BQ	$O_2^{\bullet-}$ scavenger	100:1:10:2	4	-	-	-

[a] Reaction was performed at room temperature with white LED irradiation (13 W m^{-1}, 15 mW cm^{-2}) with 10 mg Py-BPDA-COF. [b] Molecular weight and molecular weight distribution (M_w/M_n) were determined by GPC analysis (THF as eluent) calibrated with PMMA standards. [c] $M_{n,th}$ = MW (initiator) + MW (monomer) × Conv. × ([monomer]/[initiator]), where MW means molecular weight. [d] Monomer conversion was determined by ^1H NMR (Refer to Figure S11).

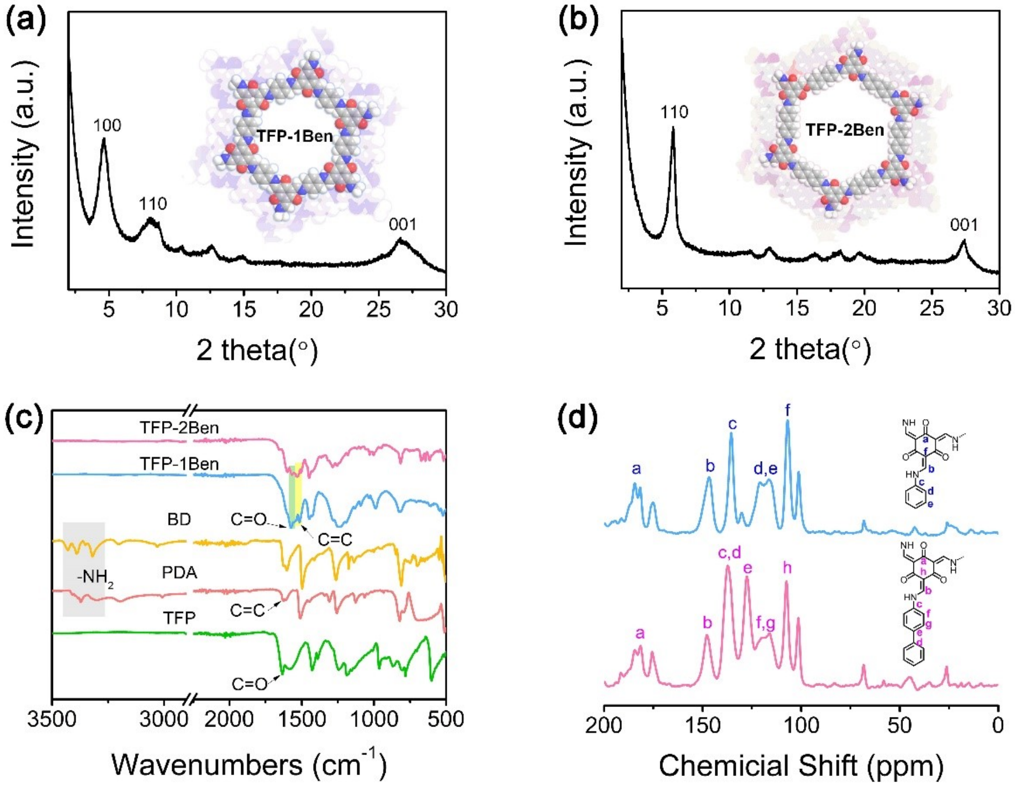

Figure S15. XRD pattern of (a) TFP-1Ben and (b) TFP-2Ben. (c) FT-IR spectrum and (d) ^{13}C CP-MAS solid state NMR spectrum of TFP-1Ben and TFP-2Ben.

The XRD pattern of TFP-1Ben presents an intense peak at 4.6°, corresponding to the (100) plane (Figure S15a). Slight peaks appear at 8.0° and 26.5°, which can be attributed to the (110) and (001) planes, respectively.[1] Similarly, the PXRD pattern of TFP-2Ben displays diffraction peaks at 5.8° and 27.4° that can be assigned to the (110) and (001) planes (Figure S15b). Moreover, in the FT-IR spectra (Figure S15c) of the two TFP COFs, the peaks at the 3300 cm^{-1} and 1628 cm^{-1} disappear, accounting for the disappearance of N-H stretching peak and the aldehyde

stretching peak in their correspondent building blocks, respectively. In parallel, the presence of the C=O stretching peak at 1570 cm^{-1} and the C=C stretching peak at 1608 cm^{-1} substantiates the condensation between amines and aldehydes and the formation of enol to keto tautomerism.[2] As per the solid-state ^{13}C CP-MAS NMR spectrum (Figure S15d), two TFP COFs show a clear signal around 184 ppm, emerging from the carbonyl carbons. Further evidence for enol to ketone tautomer formation is provided by the two peaks around 148 ppm and 106 ppm attributed to the C=C carbon.[3] The above results indicate that the successful preparation of target COFs.

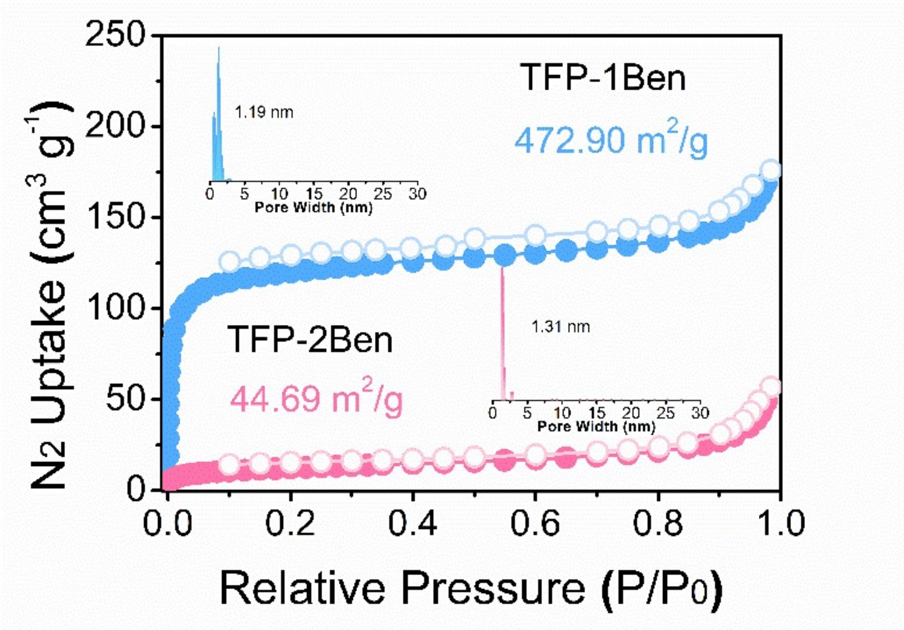

Figure S16. Nitrogen isothermal adsorption and desorption curves of (a) TFP-1Ben and (b) TFP-1Ben.

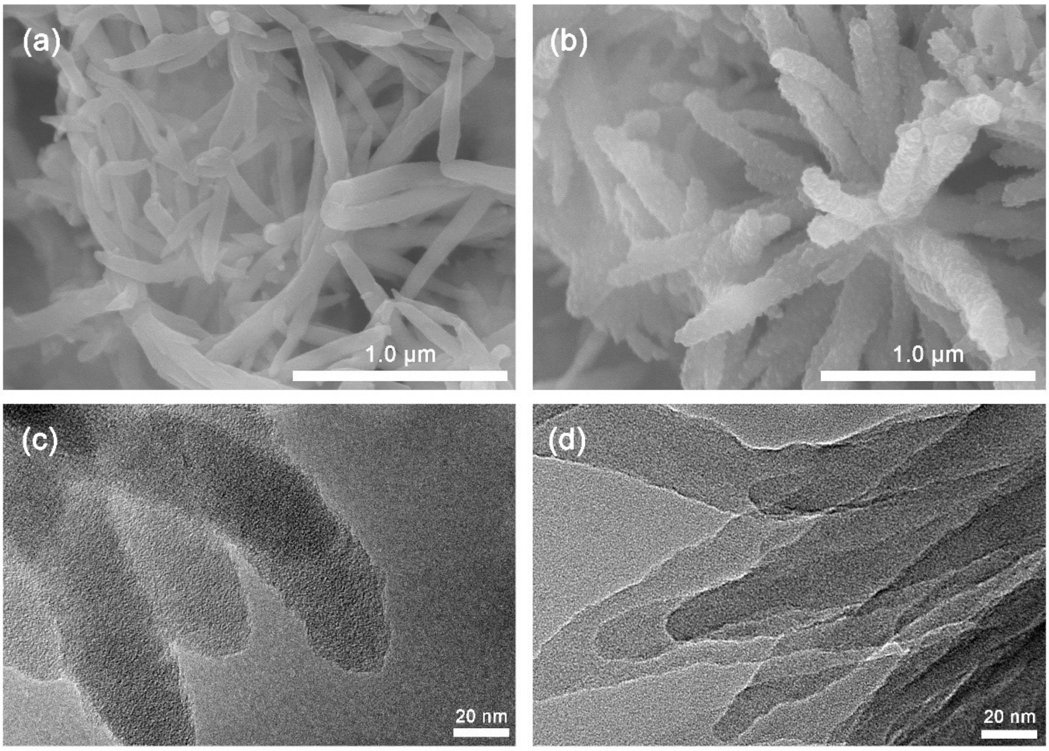

Figure S17. SEM images of (a) TFP-1Ben and (b) TFP-2Ben. TEM images of (c) TFP-1Ben and (d) TFP-2Ben.

The nitrogen adsorption/desorption isotherms of TFP COFs showed that all COFs corresponded to characteristic type I adsorption isotherms (Figure S16). The Brunauer-Emmett-Teller (BET) specific surface area estimates for TFP-1Ben and TFP-2Ben were 472.90 and 44.69 m^2 g^{-1}, respectively. According to the nonlocal density functional theory (DFT) model, the pore size distributions were estimated to be 1.19 and 1.31 nm, respectively, indicating that TFP-1Ben has a relatively smaller air gap structure. SEM and TEM images demonstrate that the COF exhibits a bar-like morphology (Figure S17).

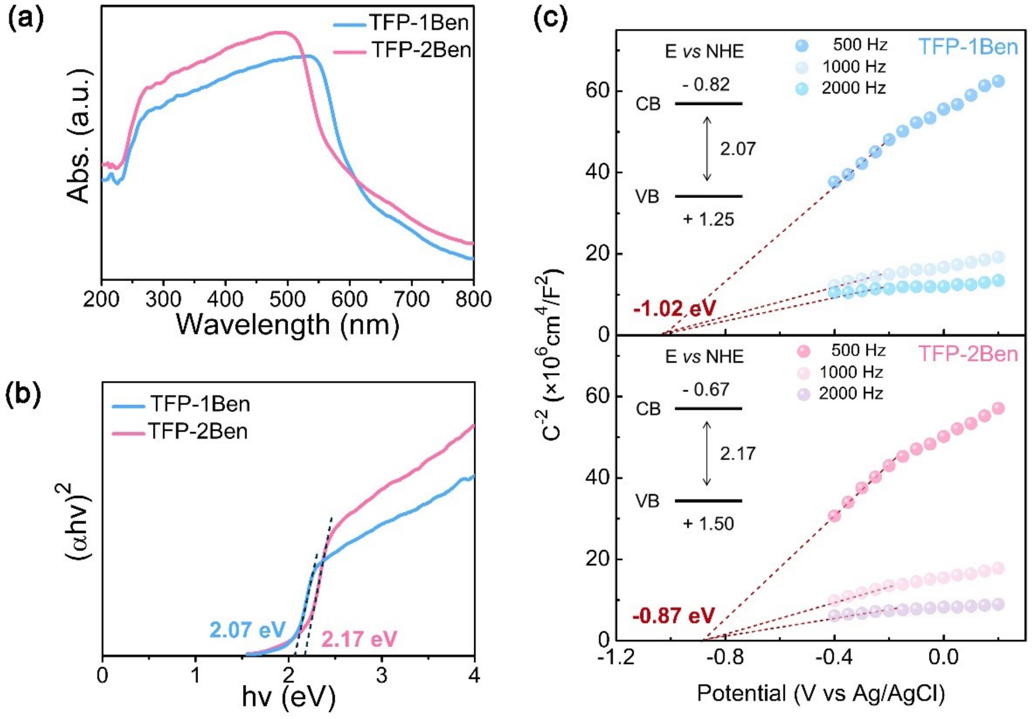

Figure S18. Photoelectrochemical measurement. (a) UV-vis DRS spectrum, (b) Tauc plots, (c) Mott-Schottky plots.

The UV-visible absorption spectra show that both TFB-1Ben and TFP-2Ben exhibit absorption in the visible range, and the calculated optical band gaps are 2.07 and 2.17 eV, respectively (Figure S18a, b). The energy band position is further evaluated by the Mott-Schottky test, and the sufficiently negative conduction band potential meets the thermodynamic requirements for the formation of superoxide anions (Figure S18c).

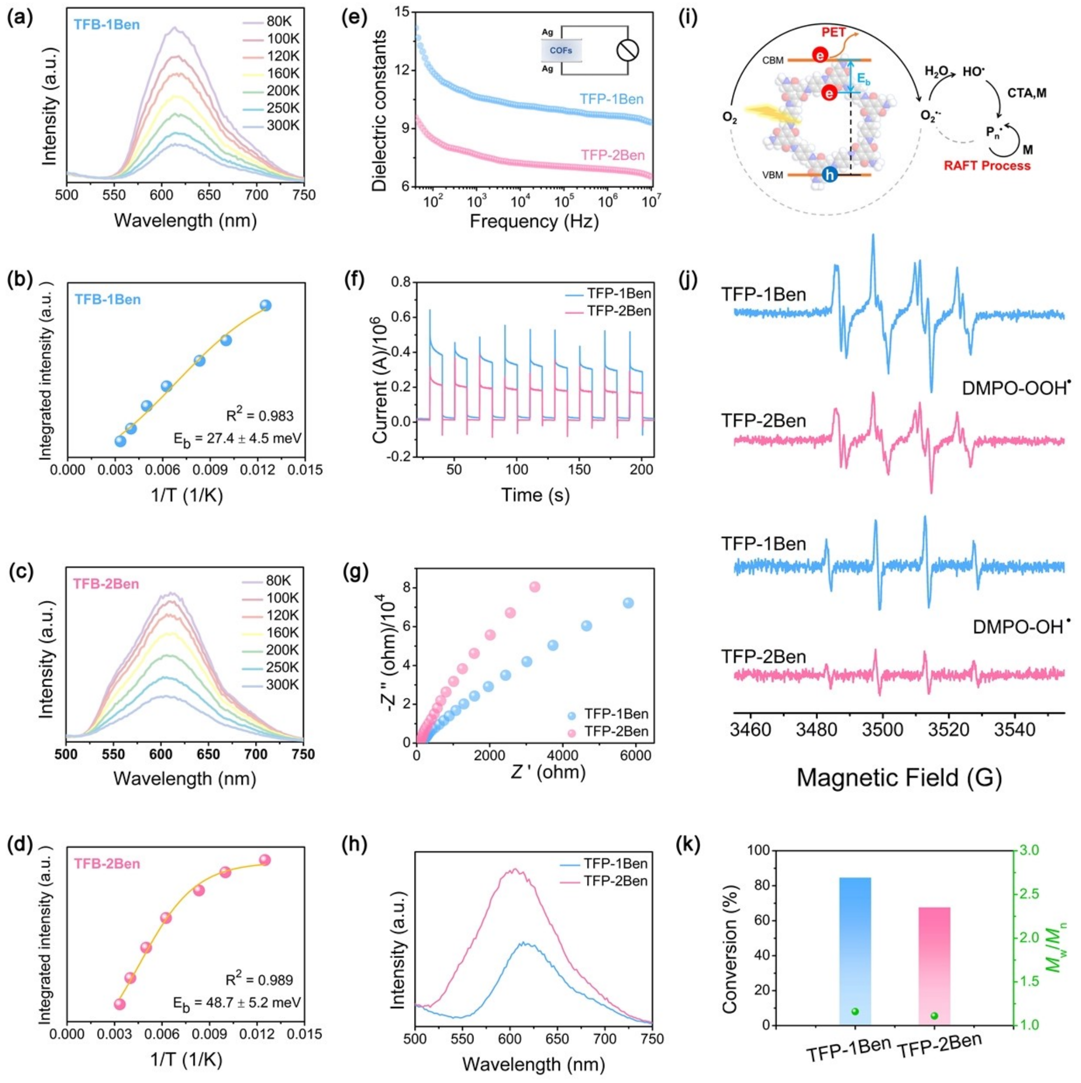

Figure S19. (a) PL spectrum versus temperature and (b) temperature inverse versus Arrhenius equation for the integrated PL emission intensity of TFP-1Ben. (c) PL spectrum versus temperature and (d) temperature inverse versus Arrhenius equation for the integrated PL emission intensity of TFP-2Ben. (e) Dielectric properties of TFP-1Ben and TFP-2Ben dependent on frequency. (f) Photocurrent response curves, (g) electrochemical impedance spectrum and (h) Steady-state fluorescence spectroscopy of TFP-1Ben and TFP-1Ben. (i) mechanism diagram of polymerization process. (j) EPR spectra. (k) Polymerization performance

comparison of TFP-1Ben and TFP-1Ben ([PEGMA]: [CPADB]: [TEA] = 100:1:10, irradiation for 6 h).

Temperature-dependent photoluminescence (PL) spectra were measured to examine the E_b. As seen from the inset of Figure S19a, c, the PL intensity of TFP COFs decreases upon raising the temperature. The temperature-dependent PL spectra were fitted by integration according to the Arrhenius equation (equation S1) to obtain the E_b. The fitting results displayed that TFP-1Ben ($E_b = 27.4 \pm 4.5$ meV) has a lower E_b than Py-BPDA-COF ($E_b = 48.7 \pm 5.2$ meV) (Figure S19b, c). The descriptor ε_r of TFP-1Ben was measured to be larger than TFP-2Ben by the parallel plate capacitor principle on an impedance analyzer. The above results show that the smaller the air gap structure, the smaller the dielectric constant of COF and the smaller its corresponding exciton binding energy when the effect of a single pore size is considered. To further verify its effect on photocatalytic activity, its photoelectric properties were evaluated. First, the photocurrent response curves showed a stronger current response signal of TFP-1Ben, implying more photogenerated electrons generation (Figure S19 f). Meanwhile, the EIS spectrum shows a smaller arc radius of TFP-1Ben, indicating its small photogenerated charge interfacial transfer resistance (Figure S19 g). More, as seen from the steady-state fluorescence spectra, the TFP-1Ben fluorescence intensity is lower, which proves that its charge-hole complexation efficiency is lower (Figure S19 h). In

summary, it can be concluded that the small air gap structure of TFB-1Ben is relatively more photocatalytically active due to its smaller E_b, which makes the splitting of excitons easier (Figure S19 i). As the superoxide anion and the hydroxyl radical, which are important substances for polymerization in the open environment, depend on the COF, the EPR spectrum shows that the TFP-1Ben with small air gap structure has a relatively stronger signal response, which corresponds to its photopolymerization activity (Figure S19 j, k).

Supplemental Experimental Procedures

Materials

All reagents and solvents were obtained from commercial outlets and used as received without further purification, except as otherwise noted. The building blocks 4,4',4'',4'''-(1,8-dihydropyrene-1,3,6,8-tetrayl)tetraaniline (PyTTA) were synthesized based on reported works[4] and terephthalaldehyde (TPA) and [1,1'-biphenyl]-4,4'-dicarbaldehyde (BPDA) were purchasing from Jilin R&S Company. 1,3,5-Triformylphloroglucinol (TFP), p-Phenylenediamine (PDA) and benzidine (BD) were purchasing from Macklin. Poly (ethylene glycol) methyl ether methacrylate ($PEGMA_{475}$, M_n = 475 g/mol), hydroxypropyl methacrylate (HPMA, 97.0%) were purchased from Aladdin and used directly without aggregation inhibitor removal. Light emitting diode (LED) strips (420-750 nm, 13 W m^{-1}, 15 mW cm^{-2}) were used as the external light source for the experiments, purchased from Philips.

Synthesis of Py-TPA-COF

Typically, PyTTA (22.8 mg,) and TPA (10.8 mg) were added into Schlenk tube, and then o-dichlorobenzene (0.5 mL), n-butanol (0.5 mL) and acetic acid (0.1 mL, 6M) served as catalyst were added. The tube was flash frozen using liquid nitrogen and then gone through freeze-pump-thaw for three times and heated at 120 °C for 3 days to afford orange-red precipitate. The product was washed with THF and soxhlet extraction with THF overnight. And the crude solid was dried under vacuum to give the final product Py-TPA-COF (~89%).

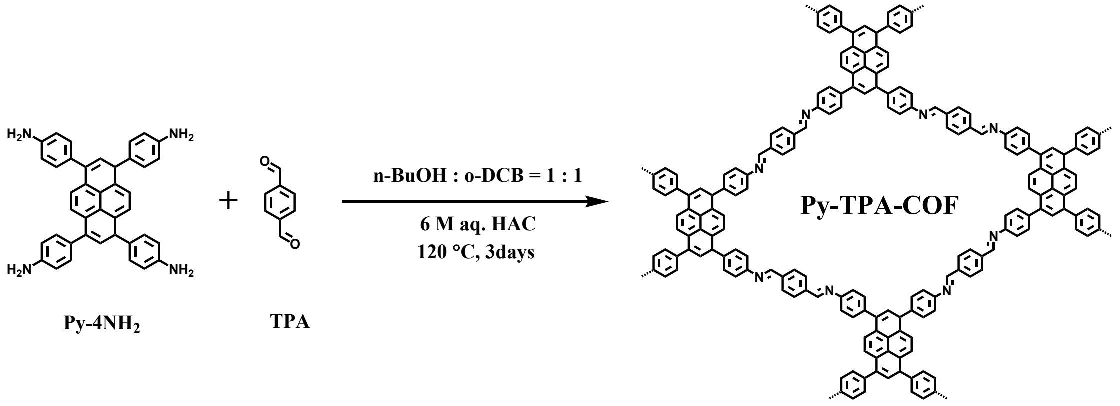

Synthesis of Py-BPDA-COF

Typically, PyTTA (22.8 mg,) and BPDA (17.0 mg) were added into Schlenk tube, and then o-dichlorobenzene (0.5 mL), n-butanol (0.5 mL) and acetic acid (0.1 mL, 6M) served as catalyst were added. The tube was flash frozen using liquid nitrogen and then gone through freeze-pump-thaw for three times and heated at 120 °C for 3 days to afford yellow precipitate. The product was washed with THF and soxhlet extraction with THF overnight. And the crude solid was dried under vacuum to give the final product Py-BPDA-COF (~75%).

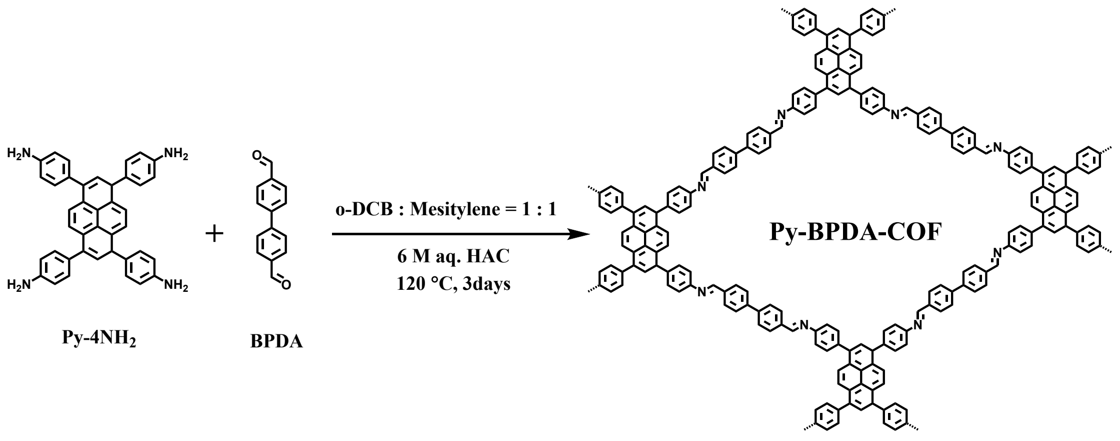

Synthesis of TFP COFs

The synthesis of TFP COFs follows the previous method.[3,5] Typically, TFP (63.0 mg, 0.3 mmol) and BDA (48.7 mg, 0.45 mmol) or BD (48.7 mg, 0.45mmol) were added into Schlenk tube, and then dioxane (1.5 mL), mesitylene (1.5 mL) and acetic acid (0.5 mL, 6M) served as catalyst were added. The tube was flash frozen using liquid nitrogen and then gone through freeze-pump-thaw for three times and heated at 120 ^{o}C for 3 days to afford yellow precipitate. The product was washed with THF and Soxhlet extraction with THF overnight. And the crude solid was dried under vacuum to give the final product TFP COFs.

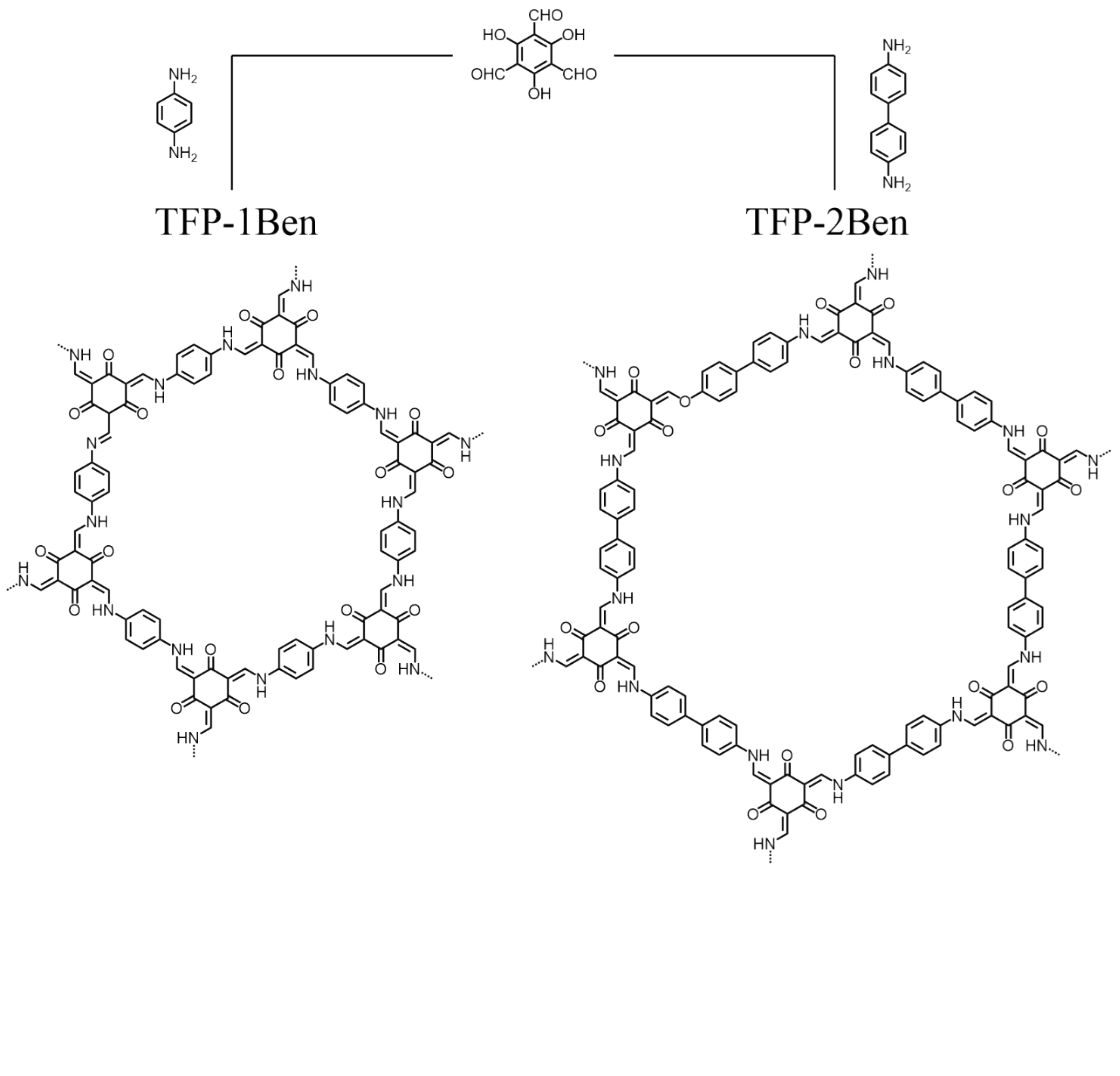

Characterization method

The powder X-ray diffraction (PXRD) spectra were recorded on DY1602/Empyrean X-ray diffractometer. Diffraction intensity data for 2 theta were collected at the scanning speed of 10 theta/min with 2 theta step increment of 0.02 theta. Fourier transform infrared (FT-IR) spectra of building blocks and COFs were recorded by Thermo Scientific Nicolet i5. ^{1}H nuclear magnetic resonance (NMR) was recorded on (14.1T/600MHz)/AVANCE NEO 600 spectrometer. ^{13}C cross-polarization magic angle rotation solid-state (CP-MAS) nuclear magnetic resonance (NMR) was recorded on Bruker 600M NMR spectrometer. UV-vis diffused reflectance spectra were measured on Shimadzu UV3600. The specific surface area and pore size distribution of the products were surveyed on an Autosorb-iQ analyzer (Quantachrome). The scanning electron microscopy (SEM, Hitachi SU8100) and transmission electron microscopy (TEM, Hitachi H-7500) were used to analyze the morphologies of COFs. Photoelectrochemical characterization was carried out on CHI660E electrochemical workstation. The steady-state photoluminescence (PL) spectra were measured under ambient condition at excitation wavelength of 365 nm on FL600 and time-resolved fluorescence (TRFL) spectra were conducted on Edinburgh FS5. Electron paramagnetic resonance (EPR) was carried out on Bruker A300. Mass spectra were logged on an Agilent 6224 Accurate Mass TOF LC/MS

spectrometer.

Gel permeation chromatography (GPC). The GPC monitoring system is fitted with a Waters 717 plus autosampler, a Waters 1515 isocratic HPLC pump, a Waters 2414 refractive index detector and Shodex K-805, K-804 and K-802.5 columns in tandem. GPC operating parameters: test flow rate: 1.0 mL/min (with THF as eluent), test temperature: 35 ^{o}C (both column and detector), calibration protocol: a series of molecular weight polymethylmethacrylate (PMMA) standards. Samples are drawn from the reaction mass at expected time intervals using a 2 mL disposable syringe and filtered through a filter to obtain a clarified sample, and the parameters (number average molecular weight (M_n) and molecular weight distribution (M_w/M_n)) of the polymer samples are determined by a GPC detection system.

Dielectric properties

An Agilent 4294A LCR impedance spectrometer was applied to examine the dielectric properties of COF powders. A thin sheet of COF powder with an electrode area of 19.625 mm^2 and a thickness of 0.45mm was pressed to form a metal-insulator-metal structure. The dielectric constant (or relative dielectric constant) reflects the polarization of the material in an electric field, and various parameters affect the intrinsic dielectric properties of the material, such as the presence of polar water and the thickness and diameter of the COF sheet. To reflect the intrinsic dielectric

constant, the thickness and diameter of the COF sheets involved were kept uniform and pre-activated in a vacuum oven at 120 °C for 24 hours to remove the water. The relative dielectric constants are generally complex values and the real part(er) can be calculated from the capacitance by the following equation S1[6]:

$$\varepsilon_r = \frac{C \cdot d}{\varepsilon_d \cdot S}$$

where C is the capacitance (F) of the COF, d is the thickness (m) of the COF sheet, ε_d is the vacuum permittivity with the value of 8.85×10^{-12} F/m, and S is the area (m^2) of the COF sheet.

Computational simulation

The P-XRD simulations were performed using a foricite module of Material Studio. The experimental XRD pattern agreed relatively well with a simulated pattern from an X-eclipsed layer stacking model, with the unit cell parameters (a = 24.0733 Å, b = 24.4929 Å, c = 3.8657 Å, α = 90°, β = 91°, γ = 90° for Py-TPA-COF and a = 28.7932 Å, b = 27.9868 Å, c = 3.7906 Å, α = 90°, β = 90°, γ = 90° for Py-BPDA-COF, respectively.) in the space group X. The density functional theory (DFT) calculations were performed using a Dmol3 module of Material Studio. The generalized gradient approximation (GGA) method with Perdew-Burke-Ernzerhof (PBE) function was employed to describe the interactions between core and electrons. The force and energy convergence criterion were set to 0.002

Ha $Å^{-1}$ and 10^{-5} Ha, respectively. When the optimization was completed, the ESP and deformation density calculations were performed. The deformed charge density subtracts the spherically symmetric charge density of each atom in the free state by the charge density of the system.

Optical band gap

UV-vis diffused reflectance spectra were measured on Shimadzu UV3600. The Tauc plot ($(\alpha h\nu)^{1/n}$ vs. $h\nu$) was converted according to the Tauc equation S2[7]:

$$(\alpha h\nu)^{1/n} = A\,(h\nu - E_g)$$

Where α represents absorption coefficient, A represents the absorption constant, and n is equal to 1/2 (direct band gap semiconductor). E_g of the samples was estimated from the intercept of the tangent in the plots of $(\alpha h\nu)^2$ versus photon energy (hν).

Photoluminescence spectra

All electrochemical related tests were made on CHI 660e electrochemical workstation which has standard three-electrode electrochemical cell with working electrode, platinum plate as counter electrode, saturated Ag/AgCl electrode as reference electrode and sodium sulfate solution (0.2 M) as electrolyte. A 300 W Xenon lamp with a 420 nm cut-off filter was used as the light source during the measurement with 10 s^{-1}switching frequency. The prepared working electrode: 5 mg of COF-based bionic enzymes

powder was blended with 2.0 mL of ethanol and 20 μL of Nafio solution and sonicated for 30 min. Afterwards, 10 μL of the mixture was dropwise placed on the surface of an FTO glass plate with an area of 1 × 1 cm^2 and placed in air to dry.

Mott-Schottky plot measurements

Mott-Schottky plot was measured in a potential range from -1.0 to 1.0 V (vs. Ag/AgCl) at a frequency of 500, 1000 and 2000 Hz without illumination. The flat band potential was obtained according to Equation S3[8]:

$$\frac{1}{C^2} = \frac{2}{N_D\, e\, \varepsilon\, \varepsilon_0}\left(E - E_{fb} - \frac{kT}{e}\right)$$

where C is the space charge capacitance in the semiconductor, N_D is the electron carrier density, e is the elemental charge, ε_0 is the permittivity of a vacuum, ε is the relative permittivity of the semiconductor, E is the applied potential, E_{fb} is the flat band potential, T is the temperature, and k is the Boltzmann constant.

The measured potentials vs. Ag/AgCl can be converted to normal hydrogen electrode (NHE) scale using Equation S4[9, 10]:

$$E(NHE) = E(Ag/AgCl) + 0.197$$

Nyquist plot measurements

Electrochemical impedance spectroscopy (EIS) was carried out on CHI 660e in a frequency range from 10^5 Hz to 10^{-2} Hz. Nyquist plot at high

frequency represents charge-transfer process and the diameter of capacitance arc reflects the charge-transfer resistance (R_{ct}).

Cyclic voltammogram measurements

Cyclic voltammogram was carried out on CHI 660e at scan rate of 0.05 V/s from 0 to - 2.0 V to + 2.0 V. Using photoluminescence maximum $E_{0,0}$ and E_{ox} / E_{red}, the excited state reduction potential was estimated for COFs according to the following Equation S5, 6[11, 12]:

$$E(PC^*/PC^{\bullet +}) = E_{ox} - E_{0,0} \text{ (Equation S4)}$$

$$E(PC^*/PC^{\bullet -}) = E_{red} + E_{0,0} \text{ (Equation S5)}$$

$OH^\bullet$ detection experiment

A 1 mL reaction mixture consisting of 0.5 mg/mL COFs and 0.09 mM MB in H_2O were added into a glass cuvette (1 mm pathlength) and sealed with a rubber septum. The mixture was irradiated under LED light (13 W m^{-1}, 15 mW cm^{-2}) and the absorbance at characteristic signals was recorded by UV-vis spectrophotometer at the indicated times.

"Water-/oxygen-fueled" PET-RAFT polymerization

Typically, $PEGMA_{475}$ (1 mL, 2.3 mmol), CPADB (6.3 mg, 0.023 mmol), H_2O (1 mL), bionic enzyme (10 mg) and TEA (32 μL, 0.23 mmol, 10 eq.) were added to a 3 mL glass vial completely exposed to air. The mixture was irradiated under white LED light (13 W m^{-1}, 15 mW cm^{-2}) at room temperature. At a predetermined time point, the LEDs are turned off and

GPC assay specimens are acquired and left in a dark environment for a period of time. Subsequently, the same GPC assay specimen is obtained before turning on the LED and illuminated for a period of time, and so forth. Samples were characterized by GPC analysis to give the number average molecular weight (M_n) and molecular weight dispersity (M_w/M_n). The monomer conversion (α) of the samples can be calculated from the corresponding peak areas of the ^{1}H NMR spectra.

In particular, when H_2O was changed to other solvents (e.g., DMAc) in the control experiments for discussing solvents, only the type of solvent was changed and not the amount added, which was 1 mL in all cases. For control experiments, scavenger (MB or BQ, the concentrations of scavenger were adjusted i.e., CPADB: scavenger = 1:0.5, 1:0.05 and 1:0.01) were added to a 3 mL glass vial completely exposed to air.

Chain extension experiments

$PEGMA_{475}$ (1 mL, 2.3 mmol), CPADB (6.3 mg, 0.023 mmol), H_2O (1 mL), bionic enzyme (10 mg), TEA (32 μL, 0.23 mmol, 10 eq.) and TEA (32 μL, 0.23 mmol, 10 eq.) were added to a 3 mL glass vial completely exposed to air. Following the monomer conversion of more than 90%, in situ chain expansion experiments were performed with DP = 150, adding the second monomer, HPMA, to the vial. After 5 h, samples were taken and characterized by GPC analysis to give the number average molecular weight (M_n) and molecular weight dispersity (M_w/M_n).

Recycle experiments

$PEGMA_{475}$ (1 mL, 2.3 mmol), CPADB (12.6 mg, 0.046 mmol), H_2O (1 mL), bionic enzyme (10 mg), and TEA (64 μL, 0.46 mmol, 10 eq.) were added to a 3 mL glass vial completely exposed to air. Bionic enzyme previously involved in aqueous RAFT polymerization of $PEGMA_{475}$ was recovered by simple centrifugation then washed with THF, dried, and reused to mediate a new aqueous RAFT polymerization of $PEGMA_{475}$ using the optimal experimental conditions.

Table S7. Fractional atomic coordinates of the Py-BPDA-COF unit cell for DFT calculations.

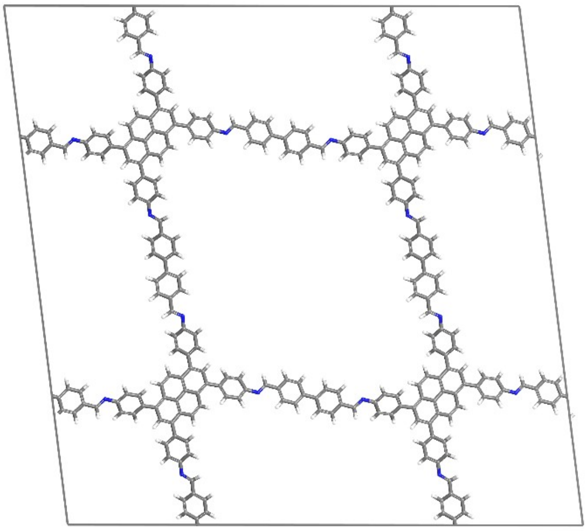

Number	Atom	X	Y	Z
1	C	45.52849	-9.55545	1.190924
2	C	46.28057	-8.65222	1.971752
3	C	46.03912	-7.28474	1.947766
4	C	44.99148	-6.74662	1.174228
5	C	44.24841	-7.63425	0.368134
6	C	44.52453	-9.00125	0.368127
7	N	44.79255	-5.3632	1.244846
8	C	43.61299	-4.85103	1.155024
9	C	43.36729	-3.41153	1.24764
10	C	44.41593	-2.48434	1.405867
11	C	44.14918	-1.13059	1.553039
12	C	42.82683	-0.62936	1.540644
13	C	41.78468	-1.56112	1.371765
14	C	42.04853	-2.92095	1.229278
15	H	47.05643	-9.03992	2.632748
16	H	46.62291	-6.60341	2.567397
17	H	43.49028	-7.24191	-0.31225
18	H	43.9856	-9.64509	-0.32708
19	H	42.70892	-5.48325	1.052334
20	H	45.43993	-2.85995	1.411871
21	H	44.98177	-0.43322	1.658819
22	H	40.74899	-1.21809	1.393424
23	H	41.21787	-3.62284	1.120134
24	C	44.78372	-15.8444	1.460973
25	C	44.34764	-14.4768	1.462584

26	C	42.94745	-14.1675	1.581568
27	C	41.99841	-15.2257	1.759402
28	C	42.48692	-16.5396	1.813595
29	C	43.83457	-16.887	1.666235
30	C	46.15399	-16.1101	1.164192
31	C	47.0745	-15.1081	1.041336
32	C	46.71528	-13.7361	1.214353
33	C	45.31859	-13.4188	1.325244
34	C	42.57438	-12.7917	1.478468
35	C	43.49572	-11.7876	1.374621
36	C	44.89463	-12.0473	1.302472
37	C	45.86499	-11.0036	1.2136
38	C	47.21715	-11.3605	1.211622
39	C	47.68018	-12.6839	1.225858
40	C	40.52801	-15.027	1.832105
41	C	39.90894	-13.8991	2.41322
42	C	38.53753	-13.6933	2.331488
43	C	37.68721	-14.6396	1.730622
44	C	38.28137	-15.8104	1.212756
45	C	39.66124	-15.9834	1.250984
46	N	36.32735	-14.3197	1.690843
47	C	35.40889	-15.217	1.57078
48	C	33.99085	-14.8649	1.51995
49	C	33.55973	-13.5309	1.665384
50	C	32.21011	-13.2151	1.630093
51	C	31.22418	-14.2085	1.441374
52	C	31.66179	-15.5405	1.302789
53	C	33.0159	-15.8618	1.341146
54	H	41.77909	-17.3432	2.015352
55	H	46.46007	-17.1418	1.004071
56	H	48.09967	-15.3556	0.77232
57	H	41.52291	-12.5222	1.478704
58	H	43.1484	-10.7568	1.3472
59	H	47.95539	-10.558	1.203985
60	H	40.50704	-13.1816	2.969689
61	H	38.08106	-12.8108	2.782036
62	H	37.66928	-16.5539	0.700408
63	H	40.09074	-16.8494	0.74536
64	H	35.6336	-16.3002	1.530524
65	H	34.31479	-12.7624	1.82829
66	H	31.90278	-12.18	1.785185
67	H	30.93021	-16.3317	1.134363
68	H	33.32839	-16.901	1.21279
69	C	49.14029	-12.9193	1.295533

70	C	49.69962	-13.9709	2.053678
71	C	51.07494	-14.1561	2.134533
72	C	51.97554	-13.2877	1.487586
73	C	51.42784	-12.212	0.753893
74	C	50.05118	-12.0436	0.6612
75	N	53.33347	-13.6116	1.598802
76	C	54.27089	-12.8198	1.199847
77	C	55.68165	-13.1891	1.275213
78	C	56.10155	-14.4306	1.792093
79	C	57.44948	-14.7545	1.843035
80	C	58.44272	-13.8586	1.387525
81	C	58.0159	-12.6202	0.870603
82	C	56.66448	-12.2932	0.816189
83	H	49.04666	-14.6231	2.632252
84	H	51.49377	-14.9674	2.730809
85	H	52.07436	-11.5429	0.184537
86	H	49.66509	-11.2406	0.032075
87	H	54.07325	-11.814	0.784582
88	H	55.34093	-15.1182	2.162279
89	H	57.75402	-15.7065	2.279672
90	H	58.75162	-11.9252	0.463827
91	H	56.3556	-11.3364	0.388395
92	C	46.8621	-27.2986	1.701818
93	C	47.69288	-26.4808	2.49252
94	C	47.43376	-25.1214	2.644639
95	C	46.335	-24.5159	2.006139
96	C	45.50517	-25.3268	1.206013
97	C	45.76203	-26.6833	1.062807
98	H	48.53079	-26.9297	3.028071
99	H	48.07607	-24.517	3.290462
100	C	46.08107	-23.0854	2.18127
101	H	44.66687	-24.854	0.692493
102	H	45.12642	-27.2793	0.405737
103	N	45.09258	-22.4912	1.601703
104	C	44.85154	-21.114	1.720151
105	C	43.73057	-20.6256	1.020515
106	C	43.41253	-19.2719	1.014757
107	C	44.20033	-18.3263	1.70239
108	C	45.30617	-18.825	2.427402
109	C	45.62594	-20.1791	2.440944
110	H	46.80192	-22.5634	2.837142
111	H	43.13837	-21.343	0.451672
112	H	42.55991	-18.9266	0.428909
113	H	45.91142	-18.1349	3.015723

114	H	46.47965	-20.5075	3.034753

Table S8. Fractional atomic coordinates of the Py-TPA-COF unit cell for DFT calculations.

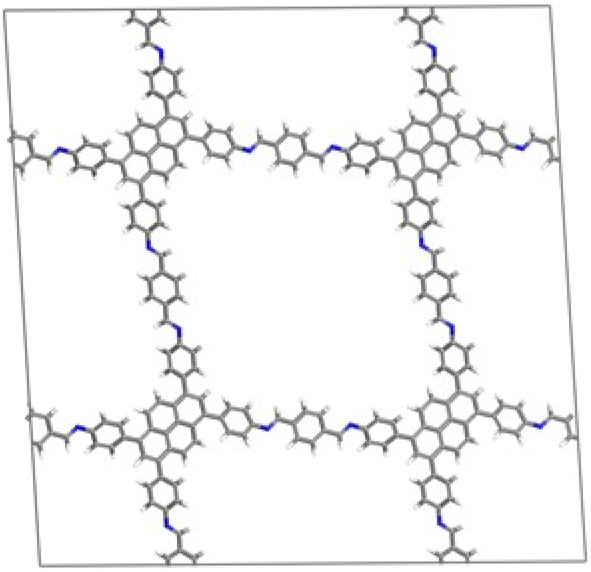

Number	Atom	X	Y	Z
1	C	16.94327	-62.1676	1.812889
2	C	16.59147	-60.7768	1.881231
3	C	15.20867	-60.3886	1.9607
4	C	14.1889	-61.3934	2.042221
5	C	14.59526	-62.737	2.033693
6	C	15.92469	-63.1594	1.916077
7	C	18.30908	-62.4997	1.562601
8	C	19.29253	-61.5513	1.540059
9	C	19.00972	-60.1696	1.775231
10	C	17.63021	-59.7742	1.848635
11	C	14.92365	-58.9878	1.909061
12	C	15.90716	-58.0388	1.894531
13	C	17.29128	-58.3786	1.876706
14	C	18.32578	-57.394	1.887432
15	C	19.65403	-57.8343	1.920837
16	C	20.03709	-59.1828	1.877333
17	C	12.73077	-61.1056	2.105808
18	C	12.17824	-59.9704	2.739689
19	C	10.82008	-59.6812	2.679736
20	C	9.911243	-60.5555	2.055066
21	C	10.43716	-61.728	1.471504

22	C	11.80652	-61.9794	1.483687
23	N	8.564286	-60.1773	2.057764
24	C	7.611879	-61.0459	2.004178
25	C	6.198074	-60.6627	2.015055
26	C	5.798357	-59.3169	2.142004
27	C	5.20034	-61.6566	1.925947
28	H	13.83241	-63.5048	2.157748
29	H	18.56325	-63.5378	1.356659
30	H	20.31279	-61.8459	1.302162
31	H	13.8907	-58.656	1.872491
32	H	15.62065	-56.9892	1.895047
33	H	20.43846	-57.0787	1.980834
34	H	12.81733	-59.3171	3.328743
35	H	10.41849	-58.7944	3.171314
36	H	9.779017	-62.4104	0.931689
37	H	12.17901	-62.8397	0.926165
38	H	7.806698	-62.1349	1.984412
39	H	6.573144	-58.5542	2.219807
40	H	5.500113	-62.7017	1.815518
41	C	15.32522	-48.9433	1.09458
42	C	16.39652	-49.5968	1.743346
43	C	17.2593	-48.8264	2.548885
44	H	14.65832	-49.5287	0.456855
45	C	16.56626	-51.0425	1.574898
46	H	18.07875	-49.3316	3.061123
47	N	17.55252	-51.6955	2.09438
48	C	17.68527	-53.0876	1.949142
49	C	18.63211	-53.7135	2.784166
50	C	18.81377	-55.0933	2.767208
51	C	18.07498	-55.9272	1.903857
52	C	17.16019	-55.29	1.034274
53	C	16.96726	-53.9109	1.052718
54	H	15.76864	-51.5346	0.988435
55	H	19.18926	-53.0856	3.480135
56	H	19.52199	-55.5428	3.464041
57	H	16.63491	-55.8859	0.287717
58	H	16.28133	-53.47	0.328506
59	C	21.4839	-59.5073	1.952406
60	C	21.97788	-60.6302	2.654968
61	C	23.33682	-60.9204	2.705828
62	C	24.28598	-60.0693	2.105556
63	C	23.80936	-58.9214	1.436482
64	C	22.44378	-58.6616	1.355323
65	N	25.6284	-60.4628	2.132833

66	C	26.58595	-59.5997	2.169973
67	C	27.99755	-59.9824	2.103163
68	C	28.39721	-61.3269	1.965373
69	C	28.99535	-58.9881	2.187196
70	H	21.28337	-61.2664	3.202127
71	H	23.69795	-61.7912	3.253007
72	H	24.51019	-58.2863	0.892557
73	H	22.10775	-57.8121	0.760466
74	H	26.39069	-58.5152	2.271468
75	H	27.62341	-62.0914	1.894708
76	H	28.69554	-57.944	2.306254
77	C	19.04346	-71.5897	2.694995
78	C	17.97313	-70.9357	2.044991
79	C	17.11296	-71.7056	1.236117
80	H	19.70655	-71.0054	3.337691
81	C	17.79271	-69.4929	2.227253
82	H	16.29269	-71.2003	0.725293
83	N	16.83007	-68.8367	1.66922
84	C	16.6764	-67.4466	1.821356
85	C	15.63457	-66.8553	1.079786
86	C	15.40411	-65.483	1.116197
87	C	16.20283	-64.6206	1.893184
88	C	17.23082	-65.2209	2.654597
89	C	17.46167	-66.5933	2.626654
90	H	18.55361	-69.0135	2.869014
91	H	15.02935	-67.5073	0.448486
92	H	14.60682	-65.059	0.504203
93	H	17.84308	-64.596	3.306264
94	H	18.25227	-67.0008	3.257847

Supplemental References

1. W. Weng and J. Guo, *Nat. Commun.*, 2022, **13**, 5768.
2. W. Wang, S. Deng, L. Ren, D. Li, W. Wang, M. Vakili, B. Wang, J. Huang, Y. Wang and G. Yu, *ACS Appl. Mater. Interfaces*, 2018, **10**, 30265-30272.
3. S. Kandambeth, A. Mallick, B. Lukose, M. V. Mane, T. Heine and R. Banerjee, *J Am. Chem. Soc.*, 2012, **134**, 19524-19527.
4. B. Dong, L. Wang, S. Zhao, R. Ge, X. Song, Y. Wang and Y. Gao, *Chem. Commun.*, 2016, **52**, 7082-7085.
5. Q. An, H.-e. Wang, G. Zhao, S. Wang, L. Xu, H. Wang, Y. Fu and H. Guo, *Energy Environ. Mater.*, 2023, **6**, e12345.
6. X. Gao, Y. Shen, J. Liu, L. Lv, M. Zhou, Z. Zhou, Y. P. Feng and L. Shen, *J Mater. Chem. C*, 2021, **9**, 15026-15033.
7. H. Ben, G. Yan, H. Liu, C. Ling, Y. Fan and X. Zhang, *Adv. Funct. Mater.*, 2022, **32**, 2104519.
8. M. Aleksandrzak, D. Baranowska, T. Kedzierski, K. Sielicki, S. Zhang, M. Biegun and E. Mijowska, *Appl. Catal. B*, 2019, **257**, 117906.
9. Q. Liu, X. He, J. Peng, X. Yu, H. Tang and J. Zhang, *Chinese J Catal.*, 2021, **42**, 1478-1487.
10. S. Niu, S. Li, Y. Du, X. Han and P. Xu, *ACS Energy Lett.*, 2020, **5**, 1083-1087.

11. X. Xu, X. Xu, Y. Zeng and F. Zhang, *J Photochem. Photobiol. A*, 2021, **411**, 113191.

12. Q. Ma, Y. Jiang, J. Lin, X. Zhang, H. Shao and S. Liao, *Polym. Chem.*, 2022, **13**, 4284-4289.
